# *luxR* Homolog-Linked Biosynthetic Gene Clusters in *Proteobacteria*

**DOI:** 10.1128/mSystems.00208-17

**Published:** 2018-03-27

**Authors:** Carolyn A. Brotherton, Marnix H. Medema, E. Peter Greenberg

**Affiliations:** a Department of Microbiology, University of Washington, Seattle, Washington, USA; b Bioinformatics Group, Wageningen University, Wageningen, The Netherlands; University of California, San Diego

**Keywords:** quorum sensing, secondary metabolism

## Abstract

Microbes are a major source of antibiotics, pharmaceuticals, and other bioactive compounds. The production of many specialized microbial metabolites is encoded in biosynthetic gene clusters (BGCs). A challenge associated with natural product discovery is that many BGCs are not expressed under laboratory growth conditions. Here we report a genome-mining approach to discover BGCs with *luxR*-type quorum sensing (QS) genes, which code for regulatory proteins that control gene expression. Our results show that BGCs linked to genes coding for LuxR-like proteins are widespread in *Proteobacteria*. In addition, we show that associations between *luxR* homolog genes and BGCs have evolved independently many times, with functionally diverse gene clusters. Overall, these clusters may provide a source of new natural products for which there is some understanding about how to elicit production.

**IMPORTANCE** Bacteria biosynthesize specialized metabolites with a variety of ecological functions, including defense against other microbes. Genes that code for specialized metabolite biosynthetic enzymes are frequently clustered together. These BGCs are often regulated by a transcription factor encoded within the cluster itself. These pathway-specific regulators respond to a signal or indirectly through other means of environmental sensing. Many specialized metabolites are not produced under laboratory growth conditions, and one reason for this issue is that laboratory growth media lack environmental cues necessary for BGC expression. Here, we report a bioinformatics study that reveals that BGCs are frequently linked to genes coding for LuxR family QS-responsive transcription factors in the phylum *Proteobacteria*. The products of these *luxR* homolog-associated gene clusters may serve as a practical source of bioactive metabolites.

## Abstract (precis)

Bacteria biosynthesize specialized metabolites with a variety of ecological functions, including defense against other microbes. Genes that code for specialized metabolite biosynthetic enzymes are frequently clustered together. These BGCs are often regulated by a transcription factor encoded within the cluster itself. These pathway-specific regulators respond to a signal or indirectly through other means of environmental sensing. Many specialized metabolites are not produced under laboratory growth conditions, and one reason for this issue is that laboratory growth media lack environmental cues necessary for BGC expression. Here, we report a bioinformatics study that reveals that BGCs are frequently linked to genes coding for LuxR family QS-responsive transcription factors in the phylum *Proteobacteria*. The products of these *luxR* homolog-associated gene clusters may serve as a practical source of bioactive metabolites.

## INTRODUCTION

Specialized bacterial metabolites have been and continue to be a major source of antibiotics and other bioactive compounds used in medicine (1, 2). The rise in antibiotic-resistant bacterial pathogens has made the discovery of novel antibiotics a pressing public health issue (3). A challenge in the field of natural-product discovery is that many biosynthetic gene clusters (BGCs) are not expressed under laboratory growth conditions (4). Although researchers have found various means to activate the expression of silent clusters, an understanding of the regulatory circuits that control BGCs of interest would provide a major practical advantage to the discovery and study of the encoded product(s) (5). Regulatory elements associated with defense from other microbes might help identify BGCs enriched in antimicrobial activity, and knowledge of the biology of those regulatory elements could guide studies aimed at “waking up” silent BGCs in laboratory culture.

We hypothesized that quorum sensing (QS)-regulated BGCs might be a rich source of novel bioactive compounds for which the regulatory components are well understood in many organisms, such as *Pseudomonas aeruginosa*. QS is a process through which bacteria sense cell density and regulate gene expression in response (6). QS allows bacteria to modify their environment in a coordinated fashion and perform cooperative metabolic activities. QS-regulated products include exopolysaccharides, extracellular enzymes, and specialized metabolites, such as antibiotics. QS-based gene regulation has been studied in many organisms, including Gram-negative and Gram-positive bacteria, and involves a variety of signal molecules (6, 7).

One type of QS is mediated by acyl-homoserine lactone (AHL) signals. In AHL QS, genes are regulated by a member of the LuxR family of transcription factors. Generally, LuxR family members bind and respond to an AHL synthesized by a homolog of the signal synthase protein LuxI (7). The AHLs can diffuse in and out of cells, and thus the concentration of AHLs within the cell serves as a proxy for cell density (8, 9). Canonical AHL QS circuits involve a LuxR family member, which responds to the specific AHL produced by a cognate LuxI family member (10). There has been relatively recent interest in a subset of LuxR-like transcription factors without a cognate LuxI-type AHL synthase. These have been termed solos or orphans. Some solos respond to AHLs produced by other bacteria, and others respond to a non-AHL-based signal produced by the bacterium harboring the LuxR-type solo or a signal from another species (11–14).

LuxR-type QS has been shown to regulate secreted metabolites with an array of ecological functions, including siderophores, redox-active molecules, and antibiotics (15–17). The LuxR-type QS-regulated products carbapenem and mupirocin are antibiotics used in clinics today (18, 19). In addition, other natural products regulated by LuxR-type QS, such as bactobolin and enacyloxin, have antibiotic activity (20, 21). In many cases where BGCs are regulated by QS, the characterized QS-regulated BGCs are located adjacent to the *luxR* gene; this is the case for the BGCs encoding mupirocin, carbapenem, bactobolin, enacyloxin, and other QS-regulated natural products discussed in more detail below (20–23). However, there are known BGCs regulated by LuxR-type regulators that are not encoded within the BGC itself but rather elsewhere in the genome, such as in the case of the pigment metabolite violacein (24).

Several known antibiotics are QS regulated, and chemical genetics methods that have been developed to study LuxR-based QS might aid in metabolite discovery and isolation by overcoming issues of BGCs that are silent under laboratory conditions. To illustrate this approach, AHL QS was first discovered to control light production in the marine bacterium *Vibrio fischeri*, where *luxI* codes for an AHL synthase and LuxR responds to the LuxI-generated AHL. Addition of *V. fischeri* AHL (3-oxohexanoyl-l-homoserine lactone) exogenously leads to early-onset and dramatically higher levels of QS-regulated bioluminescence (25). This same strategy can be used to elicit the expression of LuxR homolog-regulated BGCs, which may be silent under laboratory growth conditions due to the lack of an unknown environmental stimulus or insufficient AHL production. Another approach may involve the use of AHL-degrading lactonases, such as AiiA, which effectively degrade AHL signals. AiiA or other lactonases can be added exogenously to cultures to provide a QS-negative control without the need of doing genetic analyses (26). Finally, rich literature on LuxR-type QS provides guidance on growth conditions that activate QS in various organisms, and this literature provides a starting point for studying the expression of BGCs activated by LuxR-like proteins (27).

To fulfill the potential of this QS-based genome-mining approach, computational identification and exploration of QS-regulated BGCs is first required. Here, we performed a systematic analysis of BGCs associated with *luxR*-type genes across publicly available bacterial genome sequences. Our results show that *luxR* homolog-associated BGCs are both common and widespread in bacteria in the phylum *Proteobacteria* and that such associations have evolved multiple times within diverse BGC classes.

## RESULTS AND DISCUSSION

We used two Pfam domains that define LuxR-like proteins (Pfam accession numbers PF00196 and PF03472, which correspond to the DNA-binding and signal-binding domains, respectively) to search BGCs identified by antiSMASH and obtained a list of 2,081 *luxR* homolog-associated BGCs. We performed redundancy filtering on gene clusters that harbored LuxR homologs with greater than 90% mutual identity at the amino acid level; one representative BGC from each cluster of LuxR homologs was retained. Thus, we obtained a list of 137 BGCs (see Data Set S1 in the supplemental material).

**FIG 1:**
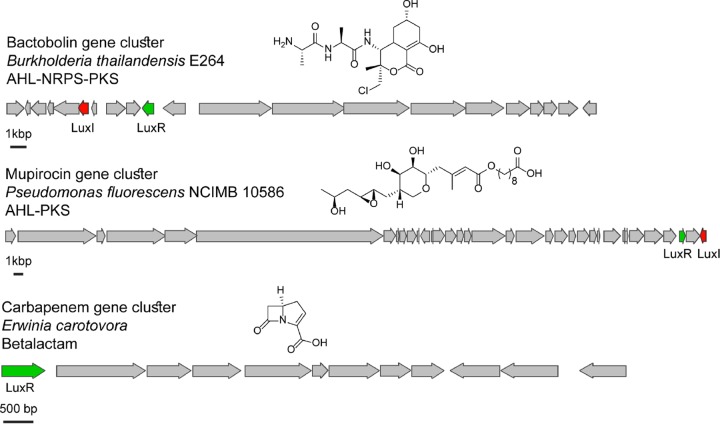
Characterized LuxR-associated and QS-regulated BGCs were identified in our screen. The *luxR* and *luxI* homologs are shown in green and red, respectively, and all other genes in each biosynthetic cluster are shown in gray. The name of the BGC, the name of the producing organism, the type of BGC, and the structure of the encoded product are shown for each example. All three of these products have potent antimicrobial activity.

Our screen identified all known, characterized *luxR* homolog-associated, QS-regulated BGCs, including those that biosynthesize bactobolin, enacyloxin, mupirocin, carbapenem, malleilactone, thailandamide, corrugation, nunamycin, and phenazine (20–23, 28–33). These clusters have been shown to be regulated by associated LuxR homolog regulators through various experimental approaches, including natural-product isolation and transcriptomics experiments with QS mutants. Representatives of these characterized hits and their corresponding products are shown in Fig. 1. To date, all known LuxR-type QS-regulated BGCs, including those listed above, are restricted to bacteria in the phylum *Proteobacteria*. These organisms were isolated from a range of sources, including plant-associated (e.g., carbapenem, produced by the plant pathogen *Erwinia carotovorum*), environmental (e.g., bactobolin, produced by soil-dwelling *Burkholderia thailandensis* E264), and host-associated (e.g., malleilactone, produced by the human-associated pathogen *Burkholderia pseudomallei*) sources. The identification of these known QS-regulated BGCs validates our bioinformatics method and confirms that this approach allows us to identify QS-regulated BGCs. These data suggest that many of the LuxR homolog-associated BGCs in our list are indeed regulated by QS systems in the producing organisms.

**FIG 2:**
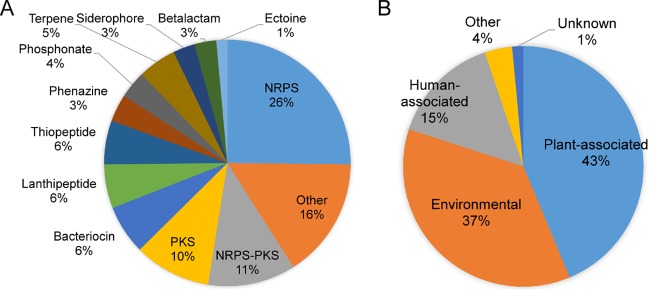
Pie charts showing the types of BGCs associated with *luxR* homologs (A) and the habitats of the organisms with *luxR* homolog-associated BGCs (B).

The types of BGCs identified in our screen were diverse (Fig. 2A). A majority of these BGCs have not been characterized, and many of the clusters likely code for the biosynthetic pathways of unknown products. The most common types of biosynthetic pathways represented were nonribosomal peptide synthetase (NRPS) (26%), polyketide synthase (PKS) (10%), and PKS-NRPS hybrid clusters (11%). Various other types of biosynthetic genes made up the remainder of the clusters, including those involved in bacteriocin, lanthipeptide, thiopeptide, terpene, siderophore, and beta-lactam biosynthesis. Forty-seven percent of the BGCs contained a gene encoding a LuxI homolog, and the remainder have no *luxI*-type gene contained within the BGC. BGCs encoding LuxI-like proteins came from every class of gene cluster type except for beta-lactam BGCs.

In terms of the ecology of these organisms, the most highly represented members of this list were plant-associated bacteria, such as *Erwinia carotovora* (Fig. 2B). We identified several BGC-associated LuxR-like proteins encoded by plant-associated bacteria that contained amino acids grouping them with the recently discovered class of LuxR homologs that respond to plant signals rather than AHL signals (34) (Fig. S1). Environmental bacteria, defined as free-living organisms isolated from soil, water, or other environmental sources, were the next most common. Finally, human-associated bacteria, for example *Acinetobacter baumannii* AYE, were the next-largest group, followed by bacteria from other habitats that did not fit into the above three categories, such as insect symbionts, like *Acetobacter malorum* strain DmCS_005.

10.1128/mSystems.00208-17.1 FIG S1  A multiple-sequence alignment reveals that several LuxR-type proteins from plant-associated bacteria identified in our screen contain amino acids that identify these sequences as likely plant-responsive regulators. The multiple-sequence alignment was performed using Clustal Omega (49). Included in this alignment are AHL-binding regulators (BjaR, *Bradyrhizobium diazoefficiens* JCM 10833 [GenBank accession number Q89VI3]; PhzR, *Pseudomonas chlororaphis* [GenBank accession number P54303]; LuxR, *Vibrio fischeri* ES114 [accession number AAW87995]; LasR, *Pseudomonas aeruginosa* PAO1 [accession number AAG04819]; TraR, *Agrobacterium tumefaciens* [accession number WP_012478148.1), plant-responsive regulators (OryR, *Xanthomonas oryzae* pv.* oryzae* KACC 10331 [GenBank accession number AAW74522.1]; NesR, *Sinorhizobium meliloti* [accession number WP_010970211]) and four putative plant-responsive LuxR-type proteins identified in our screen (accession numbers KPW40335, KDO00004, KQQ86281, and KNX78031). Numbers identify 2 amino acid residues that differentiate AHL-binding and plant-responsive LuxR proteins (34). The characterized plant-responsive LuxR family proteins OryR and NesR have an M and a W at positions 1 and 2, respectively. The M at position 1 is seen in the LuxR family protein with accession number KQQ86281. The W at position 2 is seen in all four of the putative plant-responsive regulators. Download FIG S1, PDF file, 0.2 MB. Copyright © 2018 Brotherton et al. 2018 Brotherton et al. This content is distributed under the terms of the Creative Commons Attribution 4.0 International license.

To date, LuxR-type regulators have been characterized only from bacteria within the phylum *Proteobacteria*. All but one of the BGCs identified here are encoded by bacteria in the phylum *Proteobacteria*. One hit comes from *Streptomyces canus* ATCC 12646, which is in the phylum *Actinobacteria*; its LuxR family member-associated BGC codes for biosynthesis of the antibiotic telomycin (35). According to the Pfam database, there are nine protein sequences from *Actinobacteria* that contain the LuxR signal-binding domain (Pfam accession number PF03472) (41). Although no LuxR-like protein has been characterized within *Actinobacteria*, a multiple sequence alignment of this protein with reference LuxR family sequences reveals that it differs at residues identified as amino acid positions that differentiate AHL-responsive LuxR family members and those that respond to plant-associated signals (Fig. S2) (34). The variation of amino acid residues from those in canonical, AHL-responsive as well as plant-responsive LuxR regulators suggests that this LuxR homolog may bind some novel QS signal. Researchers have isolated telomycin from broth cultures of *S. canus* ATCC 12646, which has had only its telomycin BGC sequenced; the telomycin producer *S. canus* C-509, which has had its genome fully sequenced, does not harbor a *luxI*-type gene but does harbor the *luxR* homolog containing telomycin BGC (37). Together, these data suggest that the LuxR family protein encoded in the telomycin BGC may bind and respond to a novel non-AHL signal molecule, but its role in the regulation of this antibiotic’s synthetic genes is unclear and worthy of future inquiry, especially given that no functional LuxR family protein has been characterized from any organisms in the phylum *Actinobacteria*.

10.1128/mSystems.00208-17.2 FIG S2  A multiple-sequence alignment of the LuxR-type protein encoded in the telomycin BGC from *Streptomyces canus* ATCC 12647 (GenBank accession number AKQ13286) and reference sequences suggests that the regulator from *S. canus* may bind and respond to a novel non-AHL signal molecule. The multiple-sequence alignment was performed using Clustal Omega (49). Numbers identify 2 amino acid residues that differentiate AHL-binding and plant-responsive LuxR proteins (34). The plant-responsive LuxR family proteins OryR and NesR have an M and a W at positions 1 and 2, respectively, whereas GenBank accession number AKQ13286 differs from both plant-responsive and canonical LuxR-type proteins at these two positions. Accession numbers for the reference sequences are, for TraR, NCBI accession number WP_012478148, for OryR, AAQ74522, for NesR, WP_010970211, for BjaR, Q89VI3, for LuxR, AAW87995, for PhzR, P54303, and for LasR, AAG04819. Download FIG S2, PDF file, 0.1 MB. Copyright © 2018 Brotherton et al. 2018 Brotherton et al. This content is distributed under the terms of the Creative Commons Attribution 4.0 International license.

We found that *luxR*-type QS-associated BGCs make up about 2.9% of BGCs encoded by bacteria in the phylum *Proteobacteria*; the antiSMASH database contains 72,178 BGCs encoded by *Proteobacteria*, and our unfiltered list of *luxR* homolog-associated BGCs contained 2,081 hits. We identified *luxR* family member-linked BGCs in members of four of the six classes in the phylum *Proteobacteria*. Forty-one percent of the linked BGCs were in the class *Gammaproteobacteria*, followed by the BGCs in the *Alphaproteobacteria* (31%) and *Betaproteobacteria* (26%). Interestingly, we discovered *luxR* homolog-associated BGCs in three organisms from the *Deltaproteobacteria* (*Desulfocapsa sulfexigens* DSM 10523, *Geobacter uraniireducens* Rf4, and *Haliangium ochraceum* DSM 14365). This result intrigued us, as QS has not been well studied in organisms belonging to the *Deltaproteobacteria*, and our results suggest that AHL-based QS systems may regulate specialized metabolite production in some of these organisms. We did not identify any hits in bacteria belonging to the classes *Epsilonproteobacteria*, *Oligoflexia*, and *Acidithiobacillia*. No *luxR* or *luxI* homolog genes have been identified to date from *Epsilonproteobacteria* and *Oligoflexia*. Some organisms within the *Acidithiobacillia* class, for example *Acidithiobacillus ferrooxidans* ATCC 53993, harbor *luxR-* and *luxI*-type genes, but so far none has been linked to a BGC.

We examined the genetic context of the BGCs identified in our screen. Manual examination of several BGCs showed that they had GC contents significantly different from those in the organism’s genome (Fig. S3). Altered GC content can be an indicator of horizontal gene transfer events (38). We wondered whether phylogenetic analysis of the LuxR family proteins in our list would reveal any information regarding the evolution of these proteins. We were particularly interested to see whether LuxR family proteins grouped together according to the type of BGC with which they were associated.

10.1128/mSystems.00208-17.3 FIG S3  Two representative gene clusters demonstrating different (A) and similar (B) GC contents within the *luxR* homolog-associated BGCs. The *luxR* and *luxI* homologs are shown in green and red, respectively, and all other genes in each BGC are shown in gray. As indicated in panel A, the range of the percent GC contents of the displayed region is shown, with the top number indicating the maximum percent GC content in that region, the middle number the average percent GC content, and the lower number the minimum percent GC content of that region. Download FIG S3, PDF file, 0.3 MB. Copyright © 2018 Brotherton et al. 2018 Brotherton et al. This content is distributed under the terms of the Creative Commons Attribution 4.0 International license.

Surprisingly, in the resulting phylogenetic analysis of the BGC-associated LuxR family amino acid sequences (Fig. S4), LuxR-type proteins did not group according to the type of BGC with which they were associated. We then examined the organization of the BGCs in groups of closely related LuxR-type proteins (Fig. S5). There were several examples of closely related LuxR-type proteins in which the associated LuxI family protein appeared in one BGC, but not in another. In addition, several closely related LuxR family proteins from related organisms were associated with different types of BGCs. These data suggest that the loss of the AHL synthase gene is a common evolutionary trajectory. In addition, these data suggest that LuxR homologs have dynamic associations with different types of BGCs, which have evolved independently.

10.1128/mSystems.00208-17.4 FIG S4  Neighbor-joining phylogenetic tree of all BGC-associated LuxR-type protein sequences plus reference sequences. Each entry’s label shows the accession number of the LuxR homolog, the annotation of that encoded protein, and the name of the bacterium encoding it. The color of each entry refers to the type of BGC with which that particular LuxR homolog is associated. Red, AHL-NRPS or NRPS; blue, AHL-PKS or PKS; purple, AHL-NRPS-PKS or NRPS-PKS; green, all other types of BGCs, with the type of cluster indicated next to the sequence name. Reference LuxR homolog protein sequences include BjaR (*Bradyrhizobium diazoefficiens* JCM 10833 [GenBank accession number Q89VI3]), RpaR (*Rhodopseudomonas palustris* ATCC BAA-98 [GenBank accession number Q6NCZ5]), PhzR (*Pseudomonas chlororaphis* [GenBank accession number P54303]), LuxR (*Vibrio fischeri* ES114 [accession number AAW87995]), LasR (*Pseudomonas aeruginosa* PAO1 [accession number AAG04819]), TraR (*Agrobacterium tumefaciens* [accession number WP_012478148.1]), YenR (*Yersinia enterocolitica* [GenBank accession number P54295]), EsaR (*Pantoea stewartii* [GenBank accession number P54293]), OryR (*Xanthomonas oryzae* pv.* oryzae* KACC 10331 [GenBank accession number AAW74522.1]), NesR (*Sinorhizobium meliloti* [accession number WP_010970211]), SdiA (*Escherichia coli* [accession number WP_001152715]), BraR_BtAi1 (*Burkholderia thailandensis* E264 [accession number ABC34629]), RhlR (*Pseudomonas aeruginosa* 2192 [GenBank accession number EAZ59603]), and BpsR (*Burkholderia mallei* ATCC 23344 [GenBank accession number AAS90557]). Please note that this figure can be expanded to view its details in high resolution. Download FIG S4, PDF file, 0.5 MB. Copyright © 2018 Brotherton et al. 2018 Brotherton et al. This content is distributed under the terms of the Creative Commons Attribution 4.0 International license.

10.1128/mSystems.00208-17.5 FIG S5  Closely related LuxR homologs and their associated BGCs demonstrate the dynamic associations of *luxR*-type regulator genes within BGCs. For each group of BGCs, the encoded LuxR proteins were found to be closely related and clustered together in the phylogenetic tree shown in Fig. S4. Each entry’s label shows the accession number of the LuxR homolog, the annotation of that encoded protein, and the name of the bacterium encoding it. The color of each entry refers to the type of BGC with which that particular *luxR* homolog is associated. Red, AHL-NRPS or NRPS; purple, AHL-NRPS-PKS or NRPS-PKS. Within each BGC diagram, *luxR* homologs are green, *luxI* homologs are red, and all other genes in the BGCs are purple. (A) Two *luxR* homologs from *Serratia marcescens* are associated with BGCs that are nearly identical upstream of the *luxR* homolog but differ in the loss of the *luxI* homolog and other downstream genes. (B) Clustered *luxR* homologs from various *Pseudomonas* species are associated with NRPS-type BGCs, but the *luxI* homolog in the *Pseudomonas cichorii* JBC1 BGC is absent, while it is found in the other BGCs. (C) Closely related *luxR* homologs from *Burkholderia* species show differences in the presence or absence of the *luxI* homolog as well as the type of BGC with which the *luxR* homologs are associated. Please note that this figure can be expanded to view its details in high resolution. Download FIG S5, PDF file, 2.1 MB. Copyright © 2018 Brotherton et al. 2018 Brotherton et al. This content is distributed under the terms of the Creative Commons Attribution 4.0 International license.

Back-of-the-envelope calculations suggest that the associations of *luxR* homolog genes with BGCs are not random and may reflect functional significance. For example, our data set of 219,499 BGCs included 52,433 NRPS BGCs, of which 1,019, or about 2%, were *luxR* homolog associated. In contrast, there were 2,000 ectoine BGCs in the data set, out of which only 1, or 0.05%, was LuxR associated. These data suggest that *luxR* homolog genes more commonly associate with NRPS-type BGCs than with ectoine BGCs. The predicted function(s) of the products made by ectoine (an osmolyte) and NRPS-type BGCs (often antibiotics or other defense metabolites) underlines these differences in frequency of *luxR* association.

### Conclusions.

Overall, our results suggest that *luxR* homolog-associated BGCs are common and widely distributed within bacteria belonging to the phylum *Proteobacteria*. Our data suggest that *luxR* homolog-associated BGCs are especially prevalent within plant-associated bacteria.

Based on our bioinformatics method, it is possible that the hits in our list include BGCs that are encoded near or contain a *luxR* homolog gene but are not, in fact, regulated by QS. Our method identified known LuxR-type QS-regulated BGCs, increasing our confidence that many of the hits identified in our screen are truly QS regulated. Further *in silico* analysis, such as analysis of possible LuxR-binding boxes upstream of the start codons of the biosynthetic genes, might provide additional confidence regarding the transcriptional regulation of these clusters. However, to conclude that these gene clusters are indeed regulated by QS, *in vivo* experiments will be necessary. In addition to the *luxR* homolog-associated BGCs that we identified here, there are likely other BGCs encoded within *Proteobacteria* that are regulated by LuxR-type regulators that are not encoded within the BGCs but rather elsewhere in the genome. For example, in the bacterium *Chromobacterium violaceum*, production of the pigment violacein is regulated by a LuxR-type protein that is not linked to the violacein BGC (24).

In conclusion, we anticipate that the products biosynthesized by the clusters identified here will prove to be interesting targets for further study. This is an area of active inquiry in our lab. Our list of *luxR* homolog-associated BGCs supports the view that there is still much unexplored biosynthetic chemistry within bacteria in the phylum *Proteobacteria*. Finally, the depth of understanding of the biochemical and genetic aspects of LuxR-type QS provides a practical handle on how to elicit production of the metabolites encoded by the BGCs identified here.

## MATERIALS AND METHODS

### Bioinformatic identification of LuxR-associated BGCs.

To search for BGCs with a *luxR* homolog, we queried a database of 219,499 BGCs predicted by antiSMASH v3.0 (39) across all nucleotide sequences available in GenBank (40) in December 2015. The hmmsearch tool from the HMMER package (36) was then used to search for genes encoding both Pfam (41) protein domains corresponding to LuxR (Pfam accession numbers PF00196 [DNA binding domain] and PF03472 [signal-binding domain]). BGCs predicted by antiSMASH based solely on the presence of the *luxI* homolog, itself a biosynthetic gene, were filtered out (i.e., BGCs in which the only biosynthetic gene was the *luxI* homolog signal synthase were removed from our list), leaving 2,081 hits. Out of these, 1,164 contained a *luxI* homolog (Pfam accession number PF00765). Finally, CD-HIT (42, 43) was utilized to remove redundancy by using LuxR homolog protein sequences to form homologous clusters with a sequence identity cutoff of 0.9. One LuxR sequence and its corresponding BGC were retained from each cluster, leaving 137 hits.

### Phylogenetic analysis.

LuxR homolog protein sequences were aligned with MUSCLE using a gap open penalty of −2.9, a gap extend penalty of 0, and a hydrophobicity multiplier of 1.2 (44), and the edges of the alignment were trimmed with the alignment editor of MEGA7 (45). This phylogenetic tree is shown in Fig. S4 in the supplemental material. The evolutionary history was inferred using the neighbor-joining method (46). The percentage of replicate trees in which the associated taxa clustered together in the bootstrap test (1,000 replicates) are shown next to the branches, and only bootstrap values over 50% are shown (47). The tree is drawn to scale, with branch lengths in the same units as those of the evolutionary distances used to infer the phylogenetic tree. The optimal tree with the sum of branch lengths of 29.94 is shown. The analysis involved 152 amino acid sequences. There were a total of 66 positions in the final data set. The evolutionary distances were computed using the p-distance method (48) and are in the units of the number of amino acid differences per site. All positions containing gaps and missing data were eliminated. Evolutionary analyses were conducted in MEGA7 (45). GerE (NCBI database accession number WP_000659484.1), which possesses a LuxR-like DNA binding domain (Pfam accession number PF00196) but not an AHL-binding domain, was used as an outgroup.

10.1128/mSystems.00208-17.6 DATA SET S1  List of 137 *luxR* homolog-associated BGCs identified in our bioinformatics screen. This table includes information on the name of the organism that encodes a given BGC, the nucleotide accession number for the GenBank entry in which the BGC is encoded, whether the organism’s complete genome is the nucleotide accession number provided, the BGC type (where “AHL” refers to the presence of an AHL-synthase gene), the accession number for the LuxR homolog protein, the annotation for that particular LuxR homolog, the locus tag for the *luxR* homolog gene, whether there is a *luxI* homolog encoded within the cluster, the accession number for that particular LuxI homolog, the class, order, family, and genus of the organism encoding that particular BGC, the lifestyle of the organism, and, where applicable, the name of the characterized product encoded by the BGC. Download DATA SET S1, PDF file, 0.3 MB. Copyright © 2018 Brotherton et al. 2018 Brotherton et al. This content is distributed under the terms of the Creative Commons Attribution 4.0 International license.
