# Supplementary material for: luxR Homolog-Linked Biosynthetic Gene Clusters in Proteobacteria
Source: mSystems. 2018 Mar 27;3(3):e00208-17. doi: 10.1128/mSystems.00208-17 (PMC5872303; doi:10.1128/mSystems.00208-17)
Supplement: FIG S1 [file sys003182212sf1.pdf]

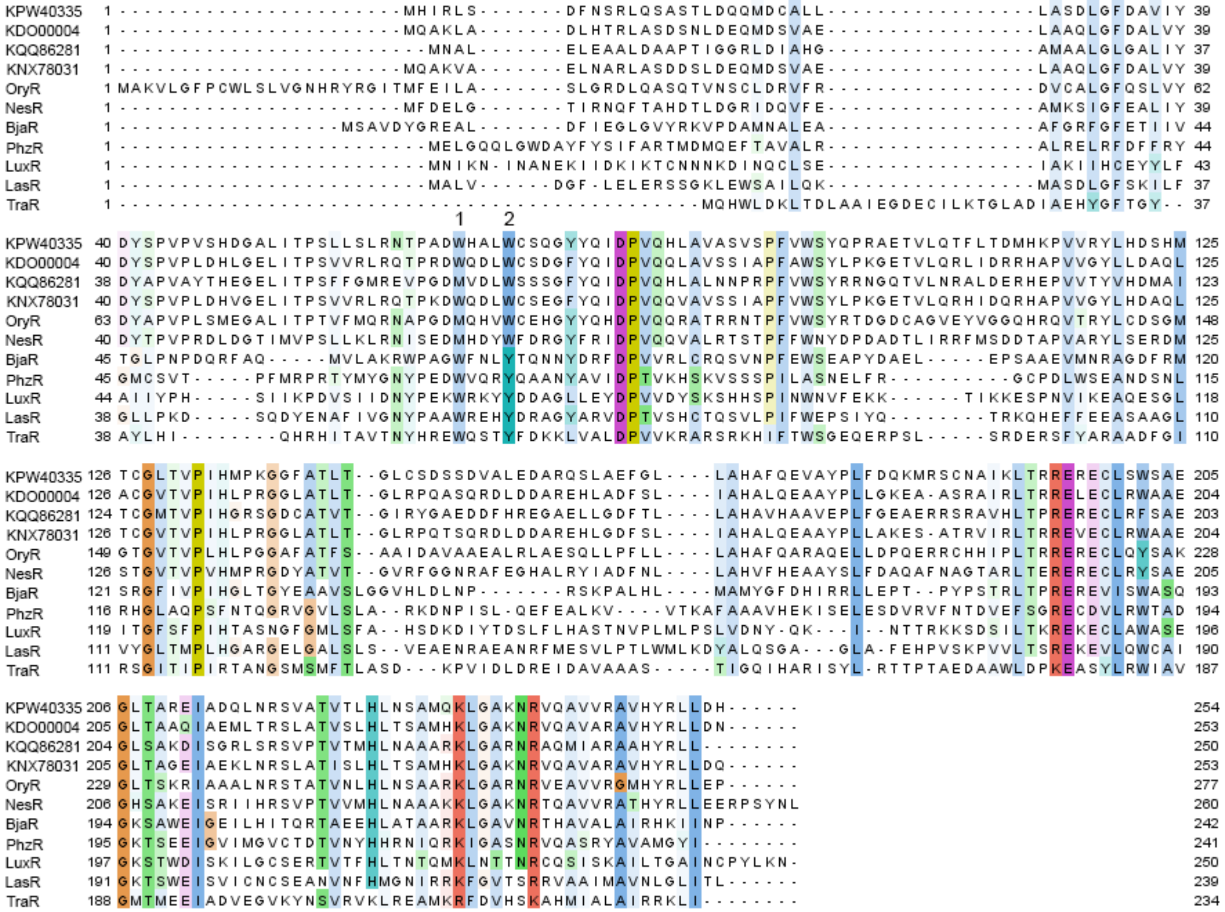
KPW40335 1 -----------------------MHIRLS-------DFNSRLQSASTLDQQMDCALL-----------------LASDLGFDAVIY 39
KDO00004 1 -----------------------MQAKLA-------DLHTRLASDSNLDEQMDSVAE-----------------LAAQLGFDALVY 39
KQQ86281 1 -------------------------MNAL-------ELEAALDAAPTIGGRLDIAHG-----------------AMAALGLGALIY 37
KNX78031 1 -----------------------MQAKVA-------ELNARLASDDSLDEQMDSVAE-----------------LAAQLGFDALVY 39
OryR 1 MAKVLGFPCWLSLVGNHRYRGITMFEILA-------SLGRDLQASQTVNSCLDRVFR-----------------DVCALGFQSLVY 62
NesR 1 -----------------------MFDELG-------TIRNQFTAHDTLDGRIDQVFE-----------------AMKSIGFEALIY 39
BjaR 1 ------------------MSAVDYGREAL-------DFIEGLGVYRKVPDAMNALEA-----------------AFGRFGFETIIV 44
PhzR 1 -------------------------MELGQQLGWDAYFYSIFARTMDMQEFTAVALR-----------------ALRELRFDFFRY 44
LuxR 1 -------------------------MNIKN-INANEKIIDKIKTCNNNKDINQCLSE-----------------IAKIIHCEYYLF 43
LasR 1 -------------------------MALV------DGF-LELERSSGKLEWSAILQK-----------------MASDLGFSKILF 37
TraR 1 ---MQHWLDKLTDLAAIEGDECILKTGLADIAEHYGFTGY-- 37
1 2
KPW40335 40 DYSPVPVSHDGALITPSLLSLRNTPADWHALWCSQGYYQIDPVQHLAVASVSPFVWSYQPRAETVLQTFLTDMHKPVVRYLHDSHM 125
KDO00004 40 DYSPVPLDHLGELITPSVVRLRQTPRDWQDLWCSDGFYQIDPVQQLAVSSIAPFAWSYLPKGETVLQRLIDRRHAPVVGYLLDAQL 125
KQQ86281 38 DYAPVAYTHEGELITPSFFGMREVPGDMVDLWSSSGFYQIDPVQHLALNNPRPFVWSYRRNGQTVLNRALDERHEPVVTYVHDMAI 123
KNX78031 40 DYSPVPLDHVGELITPSVVRLRQTPKDWQDLWCSEGFYQIDPVQQVAVSSIAPFVWSYLPKGETVLQRHIDQRHAPVVGYLHDAQL 125
OryR 63 DYAPVPLSMEGALITPTVFMQRNAPGDMQHVWCEHGYYQHDPVQQRATRRNTPFVWSYRTDGDCAGVEYVGGQHRQVTRYLCDSGM 148
NesR 40 DYTPVPRDLDGTIMVPSLLKLRNISEDMHDYWFDRGYFRIDPVQQVALRTSTPFFWNYDPDADTLIRRFMSDDTAPVARYLSERDM 125
BjaR 45 TGLPNPDQRFAQ-----MVLAKRWPAGWFNLYTQNNYDRFDPVVRLCRQSVNPFEWSEAPYDAEL-----EPSAAEVMNRAGDFRM 120
PhzR 45 GMCSVT-----PFMRPRTYMYGNYPEDWVQRYQAANYAVIDPTVKHSKVSSSPILASNELFR----------GCPDLWSEANDSNL 115
LuxR 44 AIIYPH-----SIIKPDVSIIDNYPEKWRKYYDDAGLLEYDPVVDYSKSHHSPINWNVFEKK------TIKKESPNVIKEAQESGL 118
LasR 38 GLLPKD-----SQDYENAFIVGNYPAAWREHYDRAGYARVDPTVSHCTQSVLPIFWEPSIYQ--------TRKQHEFFEEASAAGL 110
TraR 38 AYLHI--------QHRHITAVTNYHREWQSTYFDKKLVALDPVVKRARSRKHIFTWSGEQERPSL-----SRDERSFYARAADFGI 110
KPW40335 126 TCGLTVPIHMPKGGFATLT--GLCSDSSDVALEDARQSLAEFGL----LAHAFQEVAYPLFDQKMRSCNAIKLTRRERECLSWSAE 205
KDO00004 126 ACGVTVPIHLPRGGLATLT--GLRPQASQRDLDDAREHLADFSL----IAHALQEAAYPLLGKEA-ASRAIRLTRRELECLRWAAE 204
KQQ86281 124 TCGMTVPIHGRSGDCATVT--GIRYGAEDDFHREGAELLGDFTL----LAHAVHAAVEPLFGEAERRSRAVHLTPRERECLRFSAE 203
KNX78031 126 TCGVTVPIHLPRGGLATLT--GLRPQTSQRDLDDAREHLGDFSL----IAHALQEAAYPLLAKES-ATRVIRLTRREVECLRWAAE 204
OryR 149 GTGVTVPLHLPGGAFATFS--AAIDAVAAEALRLAESQLLPFLL----LAHAFQARAQELLDPQERRCHHIPLTRRERECLQYSAK 228
NesR 126 STGVTVPVHMPRGDYATVT--GVRFGGNRAFEGHALRYIADFNL----LAHVFHEAAYSLFDAQAFNAGTARLTERERECLRYSAE 205
BjaR 121 SRGFIVPIHGLTGYEAAVSLGGVHLDLNP-------RSKPALHL----MAMYGFDHIRRLLEPT--PYPSTRLTPREREVISWASQ 193
PhzR 116 RHGLAQPSFNTQGRVGVLSLA--RKDNPISL-QEFEALKV----VTKAFAAAVHEKISELESDVRVFNTDVEFSGRECDVLRWTAD 194
LuxR 119 ITGFSFPIHTASNGFGMLSFA--HSDKDIYTDSLFLHASTNVPLMLPSLVDNY-QK---I--NTTRKKSDSILTKREKECLAWASE 196
LasR 111 VYGLTMPLHGARGELGALSLS--VEAENRAEANRFMESVLPTLWMLKDYALQSGA---GLA-FEHPVSKPVVLTSREKEVLQWCAI 190
TraR 111 RSGITIPIRTANGSMSMFTLASD---KPVIDLDREIDAVAAAS-----TIGQIHARISYL-RTTPTAEDAAWLDPKEASYLRWIAV 187
KPW40335 206 GLTAREIADQLNRSVATVTLHLNSAMQKLGAKNRVQAVVRAVHYRLLDH------ 254
KDO00004 205 GLTAAQIAEMLTRSLATVSLHLTSAMHKLGAKNRVQAVARAVHYRLLDN------ 253
KQQ86281 204 GLSAKDISGRLSRSVPTVTMHLNAAARKLGARNRAQMIARAAHYRLL-------- 250
KNX78031 205 GLTAGEIAEKLNRSLATISLHLTSAMHKLGAKNRVQAVARAVHYRLLDQ------ 253
OryR 229 GLTSKRIAAALNRSTATVNLHLNSAARKLGARNRVEAVVRGMHYRLLEP------ 277
NesR 206 GHSAKEISRIIHRSVPTVVMHLNAAAKKLGAKNRTQAVVRATHYRLLEERPSYNL 260
BjaR 194 GKSAWEIGEILHITQRTAEEHLATAARKLGAVNRTHAVALAIRHKIINP------ 242
PhzR 195 GKTSEEIGVIMGVCTDTVNYHHRNIQRKIGASNRVQASRYAVAMGYI-------- 241
LuxR 197 GKSTWDISKILGCSERTVTFHLTNTQMKLNTTNRCQSISKAILTGAINCPYLKN- 250
LasR 191 GKTSWEISVICNCSEANVNFHMGNIRRKFGVTSRRVAAIMAVNLGLITL------ 239
TraR 188 GMTMEEIADVEGVKYNSVRVKLREAMKRFDVHSKAHMIALAIRRKLI-------- 234
