# Supplementary material for: luxR Homolog-Linked Biosynthetic Gene Clusters in Proteobacteria
Source: mSystems. 2018 Mar 27;3(3):e00208-17. doi: 10.1128/mSystems.00208-17 (PMC5872303; doi:10.1128/mSystems.00208-17)
Supplement: FIG S2 [file sys003182212sf2.pdf]

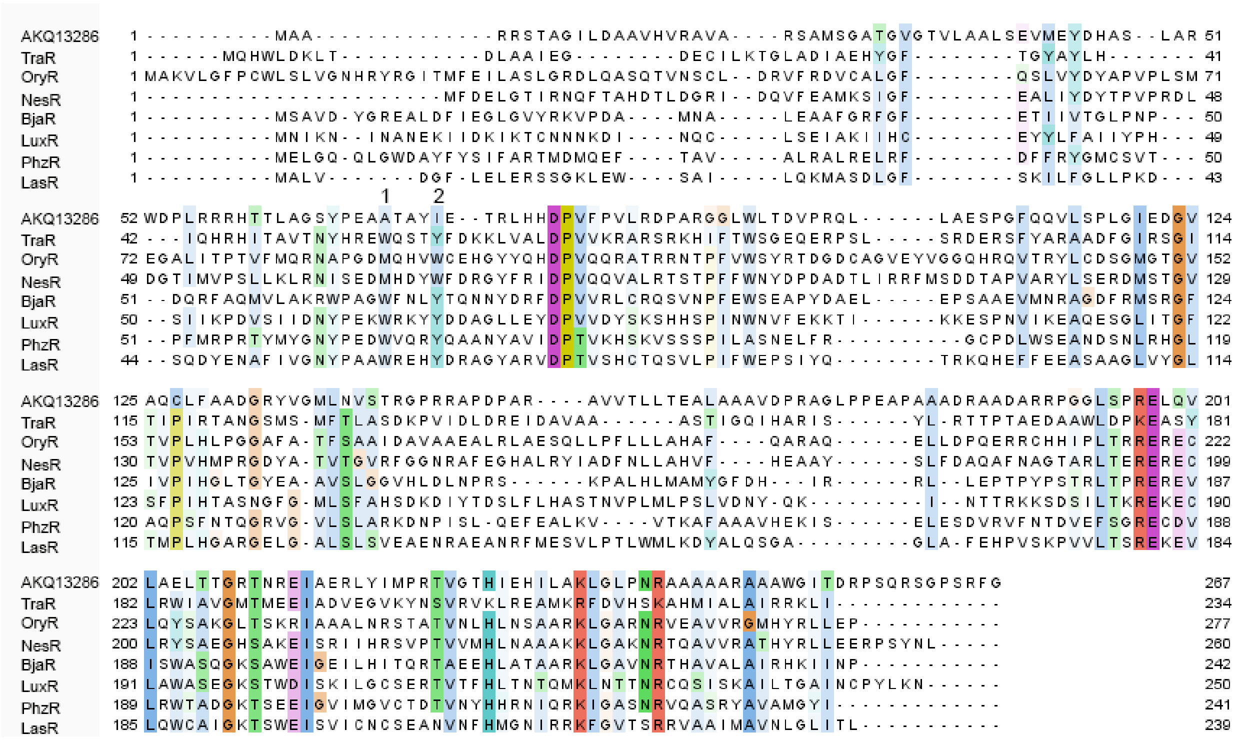
AKQ13286 1 ----------MAA--------------RRSTAGILDAAVHVRAVA----RSAMSGATGVGTVLAALSEVMEYDHAS--LAR 51
TraR 1 ------MQHWLDKLT-----------DLAAIEG--------DECILKTGLADIAEHYGF--------TGYAYLH------- 41
OryR 1 MAKVLGFPCWLSLVGNHRYRGITMFEILASLGRDLQASQTVNSCL--DRVFRDVCALGF--------QSLVYDYAPVPLSM 71
NesR 1 -----------------------MFDELGTIRNQFTAHDTLDGRI--DQVFEAMKSIGF--------EALIYDYTPVPRDL 48
BjaR 1 ----------MSAVD-YGREALDFIEGLGVYRKVPDA----MNA-----LEAAFGRFGF--------ETIIVTGLPNP--- 50
LuxR 1 ----------MNIKN--INANEKIIDKIKTCNNNKDI----NQC-----LSEIAKIIHC--------EYYLFAIIYPH--- 49
PhzR 1 ----------MELGQ-QLGWDAYFYSIFARTMDMQEF----TAV-----ALRALRELRF--------DFFRYGMCSVT--- 50
LasR 1 ----------MALV-------DGF-LELERSSGKLEW----SAI-----LQKMASDLGF--------SKILFGLLPKD--- 43
1
2
AKQ13286 52 WDPLRRRHTTLAGSYPEAATAYIE--TRLHHDPVFPVLRDPARGGLWLTDVPRQL------LAESPGFQQVLSPLGIEDGV 124
TraR 42 ---IQHRHITAVTNYHREWQSTYFDKKLVALDPVVKRARSRKHIFTWSGEQERPSL-----SRDERSFYARAADFGIRSGI 114
OryR 72 EGALITPTVFMQRNAPGDMQHVWCEHGYYQHDPVQQRATRRNTPFVWSYRTDGDCAGVEYVGGQHRQVTRYLCDSGMGTGV 152
NesR 49 DGTIMVPSLLKLRNISEDMHDYWFDRGYFRIDPVQQVALRTSTPFFWNYDPADTLIRRFMSDDTAPVARYLSERDMSTGV 129
BjaR 51 --DQRFAQMVLAKRWPAGWFNLYTQNNYDRFDPVVRLCRQSVNPFEWSEAPYDAEL-----EPSAAEVMNRAGDFRMSRGF 124
LuxR 50 --SIIKPDVSIIDNYPEKWRKYYDDAGLLEYDPVVDYSKSHHSPINWNVFEKKTI------KKESPNVIKEAQESGLITGF 122
PhzR 51 --PFMRPRTYMYGNYPEDWVQRYQAANYAVIDPTVKHSKVSSSPILASNELFR----------GCPDLWSEANDSNLRHGL 119
LasR 44 --SQDYENAFIVGNYPAAWREHYDRAGYARVDPTVSHCTQSVLPIFWEPSIYQ--------TRKQHEFFEEASAAGLVYGL 114
AKQ13286 125 AQCLFAADGRYVGMLNVSTRGPRRAPDPAR----AVVTLLTEALAAAVDPRAGLPPEAPAAADRAADARRPGGLSPRELQV 201
TraR 115 TIPIRTANGSMS-MFTLASDKPVIDLDREIDAVAA------ASTIGQIHARIS------YL-RTTPTAEDAAWLDPKEASY 181
OryR 153 TVPLHLPGGAFA-TFSAAIDAVAAEALRLAESQLLPFLLLAHAF----QARAQ------ELLDPQERRCHHIPLTRREREC 222
NesR 130 TVPVHMPRGDYA-TVTGVRFGGNRAFEGHALRYIADFNLLAHVF----HEAAY------SLFDAQAFNAGTARLTEREREC 199
BjaR 125 IVPIHGLTGYEA-AVSLGGVHLDLNPRS------KPALHLMAMYGFDH---IR------RL--LEPTPYPSTRLTPREREV 187
LuxR 123 SFPIHTASNGFG-MLSFAHSDKDIYTDSLFLHASTNVPLMLPSLVDNY-QK---------I--NTTRKKSDSILTKREKEC 190
PhzR 120 AQPSFNTQGRVG-VLSLARKDNPISL-QEFEALKV----VTKAFAAAVHEKIS------ELESDVRVFNTDVEFSGRECDV 188
LasR 115 TMPLHGARGELG-ALSLSVEAENRAEANRFMESVLPTLWMLKDYALQSGA---------GLA-FEHPVSKPVVLTSREKEV 184
AKQ13286 202 LAELTTGRTNREIAERLYIMPRTVGTHIEHILAKLGLPNRAAAAARAAAWGITDRPSQRSGPSRFG 267
TraR 182 LRWIAVGMTMEEIADVEGVKYNSVRVKLREAMKRFDVHSKAHMIALAIRRKLI------------- 234
OryR 223 LQYSAKGLTSKRIAAALNRSTATVNLHLNSAARKLGARNRVEAVVRGMHYRLLEP----------- 277
NesR 200 LRYSAEGHSAKEISRIIHRSVPTVVMHLNAAAKKLGAKNRTQAVVRATHYRLLEERPSYNL----- 260
BjaR 188 ISWASQGKSAWEIGEILHITQRTAEEHLATAARKLGAVNRTHAVALAIRHKIINP----------- 242
LuxR 191 LAWASEGKSTWDISKILGCSERTVTFHLTNTQMKLNTTNRCQSISKAILTGAINCPYLKN------ 250
PhzR 189 LRWTADGKTSEEIGVIMGVCTDTVNYHHRNIQRKIGASNRVQASRYAVAMGYI------------- 241
LasR 185 LQWCAIGKTSWEISVICNCSEANVNFHMGNIRRKFGVTSRRVAAIMAVNLGLITL----------- 239
