# Supplementary material for: luxR Homolog-Linked Biosynthetic Gene Clusters in Proteobacteria
Source: mSystems. 2018 Mar 27;3(3):e00208-17. doi: 10.1128/mSystems.00208-17 (PMC5872303; doi:10.1128/mSystems.00208-17)
Supplement: FIG S3 [file sys003182212sf3.pdf]

A

Organism: Escherichia coli KTE67
Displayed genomic region: Locus tags A1U7_02817-A1U7_02824
Average GC content of BGC (shown): 39.32%
Genomic DNA GC content: 50.48%

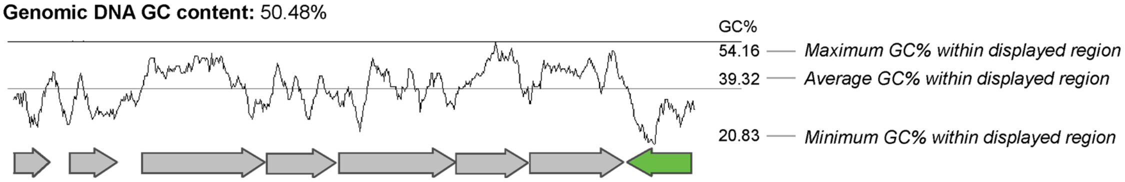

B

Organism: Acinetobacter baumanii AYE
Displayed genomic region: Locus tags ABAYE3750-ABAYE3761
Average GC content of BGC (shown): 41.45%
Genomic DNA GC content: 39.32%

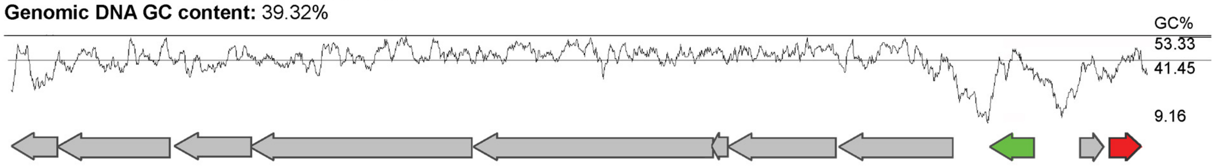

References:
