# Supplementary material for: luxR Homolog-Linked Biosynthetic Gene Clusters in Proteobacteria
Source: mSystems. 2018 Mar 27;3(3):e00208-17. doi: 10.1128/mSystems.00208-17 (PMC5872303; doi:10.1128/mSystems.00208-17)
Supplement: FIG S5 [file sys003182212sf5.pdf]

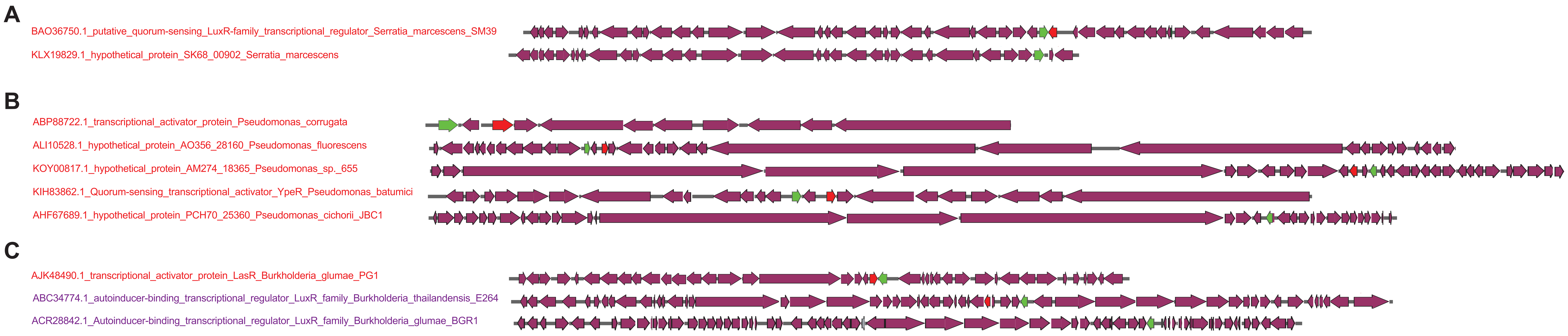
A
BAO36750.1_putative_quorum-sensing_LuxR-family_transcriptional_regulator_Serratia_marcescens_SM39
KLX19829.1_hypothetical_protein_SK68_00902_Serratia_marcescens
B
ABP88722.1_transcriptional_activator_protein_Pseudomonas_corrugata
ALI10528.1_hypothetical_protein_AO356_28160_Pseudomonas_fluorescens
KOY00817.1_hypothetical_protein_AM274_18365_Pseudomonas_sp._655
KIH83862.1_Quorum-sensing_transcriptional_activator_YpeR_Pseudomonas_batumici
AHF67689.1_hypothetical_protein_PCH70_25360_Pseudomonas_cichorii_JBC1
C
AJK48490.1_transcriptional_activator_protein_LasR_Burkholderia_glumae_PG1
ABC34774.1_autoinducer-binding_transcriptional_regulator_LuxR_family_Burkholderia_thailandensis_E264
ACR28842.1_Autoinducer-binding_transcriptional_regulator_LuxR_family_Burkholderia_glumae_BGR1
