# Supplementary material for: luxR Homolog-Linked Biosynthetic Gene Clusters in Proteobacteria
Source: mSystems. 2018 Mar 27;3(3):e00208-17. doi: 10.1128/mSystems.00208-17 (PMC5872303; doi:10.1128/mSystems.00208-17)
Supplement: FIG S4 [file sys003182212sf4.pdf]

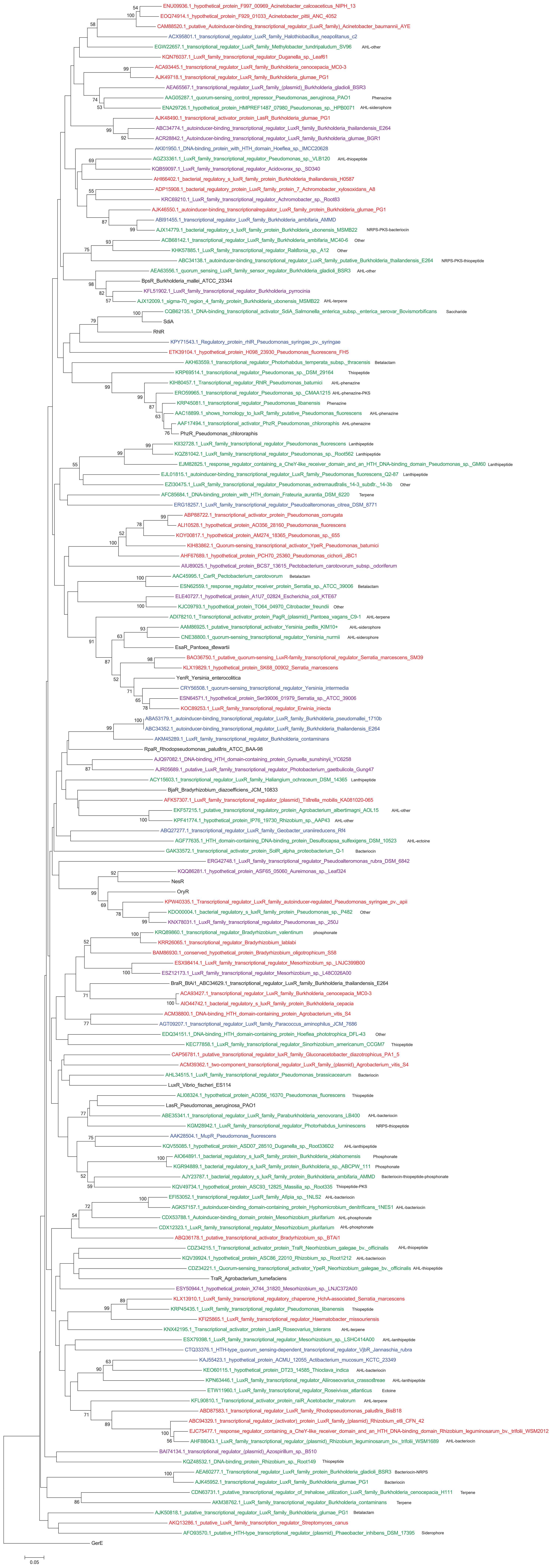
ENU09936.1_hypothetical_protein_F997_00969_Acinetobacter_calcoaceticus_NIPH_13
EOQ74914.1_hypothetical_protein_F929_01033_Acinetobacter_pittii_ANC_4052
CAM88520.1_putative_Autoinducer-binding_transcriptional_regulator_(LuxR_family)_Acinetobacter_baumannii_AYE
ACX95801.1_transcriptional_regulator_LuxR_family_Halothiobacillus_neapolitanus_c2
EGW22657.1_transcriptional_regulator_LuxR_family_Methylobacter_tundripaludum_SV96 AHL-other
KQN76037.1_LuxR_family_transcriptional_regulator_Duganella_sp._Leaf61
ACA93445.1_transcriptional_regulator_LuxR_family_Burkholderia_cenocepacia_MC0-3
AJK49718.1_transcriptional_regulator_LuxR_family_Burkholderia_glumae_PG1
AEA65567.1_transcriptional_regulator_LuxR_family_(plasmid)_Burkholderia_gladioli_BSR3
AAG05287.1_quorum-sensing_control_repressor_Pseudomonas_aeruginosa_PAO1 Phenazine
ENA29726.1_hypothetical_protein_HMPREF1487_07980_Pseudomonas_sp._HPB0071 AHL-siderophore
AJK48490.1_transcriptional_activator_protein_LasR_Burkholderia_glumae_PG1
ABC34774.1_autoinducer-binding_transcriptional_regulator_LuxR_family_Burkholderia_thailandensis_E264
ACR28842.1_Autoinducer-binding_transcriptional_regulator_LuxR_family_Burkholderia_glumae_BGR1
AKI01950.1_DNA-binding_protein_with_HTH_domain_Hoeflea_sp._IMCC20628
AGZ33361.1_LuxR_family_transcriptional_regulator_Pseudomonas_sp._VLB120 AHL-thiopeptide
KQB59097.1_LuxR_family_transcriptional_regulator_Acidovorax_sp._SD340
AHI66402.1_bacterial_regulatory_s_luxR_family_protein_Burkholderia_thailandensis_H0587
ADP15908.1_bacterial_regulatory_protein_LuxR_family_protein_7_Achromobacter_xylosoxidans_A8
KRC69210.1_LuxR_family_transcriptional_regulator_Achromobacter_sp._Root83
AJK46550.1_autoinducer-binding_transcriptionalregulator_LuxR_family_protein_Burkholderia_glumae_PG1
ABI91455.1_transcriptional_regulator_LuxR_family_Burkholderia_ambifaria_AMMD
AJX14779.1_bacterial_regulatory_s_luxR_family_protein_Burkholderia_ubonensis_MSMB22 NRPS-PKS-bacteriocin
ACB68142.1_transcriptional_regulator_LuxR_family_Burkholderia_ambifaria_MC40-6 Other
KHK57885.1_LuxR_family_transcriptional_regulator_Ralstonia_sp._A12 Other
ABC34138.1_autoinducer-binding_transcriptional_regulator_LuxR_family_putative_Burkholderia_thailandensis_E264 NRPS-PKS-thiopeptide
AEA63556.1_quorum_sensing_LuxR_family_sensor_regulator_Burkholderia_gladioli_BSR3 AHL-other
BpsR_Burkholderia_mallei_ATCC_23344
KFL51902.1_LuxR_family_transcriptional_regulator_Burkholderia_pyrrocinia
AJX12009.1_sigma-70_region_4_family_protein_Burkholderia_ubonensis_MSMB22 AHL-terpene
CQB62135.1_DNA-binding_transcriptional_activator_SdiA_Salmonella_enterica_subsp._enterica_serovar_Bovismorbificans Saccharide
SdiA
RhlR
KPY71543.1_Regulatory_protein_rhlR_Pseudomonas_syringae_pv._syringae
ETK39104.1_hypothetical_protein_H098_23930_Pseudomonas_fluorescens_FH5
AKH63559.1_transcriptional_regulator_Photorhabdus_temperata_subsp._thracensis Betalactam
KRP69514.1_transcriptional_regulator_Pseudomonas_sp._DSM_29164 Thiopeptide
KIH80457.1_Transcriptional_regulator_RhlR_Pseudomonas_batumici AHL-phenazine
ERO59965.1_transcriptional_regulator_Pseudomonas_sp._CMAA1215 AHL-phenazine-PKS
KRP45081.1_transcriptional_regulator_Pseudomonas_libanensis Phenazine
AAC18899.1_shows_homology_to_luxR_family_putative_Pseudomonas_fluorescens AHL-phenazine
AAF17494.1_transcriptional_activator_PhzR_Pseudomonas_chlororaphis AHL-phenazine
PhzR_Pseudomonas_chlororaphis
KII32728.1_LuxR_family_transcriptional_regulator_Pseudomonas_fluorescens Lanthipeptide
KQZ61042.1_LuxR_family_transcriptional_regulator_Pseudomonas_sp._Root562 Lanthipeptide
EJM82825.1_response_regulator_containing_a_CheY-like_receiver_domain_and_an_HTH_DNA-binding_domain_Pseudomonas_sp._GM60 Lanthipeptide
EJL01815.1_autoinducer-binding_transcriptional_regulator_LuxR_family_Pseudomonas_fluorescens_Q2-87 Lanthipeptide
EZI30475.1_LuxR_family_transcriptional_regulator_Pseudomonas_extremaustralis_14-3_substr._14-3b Other
AFC85684.1_DNA-binding_protein_with_HTH_domain_Frateuria_aurantia_DSM_6220 Terpene
ERG18257.1_LuxR_family_transcriptional_regulator_Pseudoalteromonas_citrea_DSM_8771
ABP88722.1_transcriptional_activator_protein_Pseudomonas_corrugata
ALI10528.1_hypothetical_protein_AO356_28160_Pseudomonas_fluorescens
KOY00817.1_hypothetical_protein_AM274_18365_Pseudomonas_sp._655
KIH83862.1_Quorum-sensing_transcriptional_activator_YpeR_Pseudomonas_batumici
AHF67689.1_hypothetical_protein_PCH70_25360_Pseudomonas_cichorii_JBC1
AIU89025.1_hypothetical_protein_BCS7_13615_Pectobacterium_carotovorum_subsp._odoriferum
AAC45995.1_CarR_Pectobacterium_carotovorum Betalactam
ESN62559.1_response_regulator_receiver_protein_Serratia_sp._ATCC_39006 Betalactam
ELE40727.1_hypothetical_protein_A1U7_02824_Escherichia_coli_KTE67
KJC09793.1_hypothetical_protein_TO64_04970_Citrobacter_freundii Other
ADI78210.1_Transcriptional_activator_protein_PagR_(plasmid)_Pantoea_vagans_C9-1 AHL-terpene
AAM86925.1_putative_transcriptional_activator_Yersinia_pestis_KIM10+ AHL-siderophore
CNE38800.1_quorum-sensing_transcriptional_regulator_Yersinia_nurmii AHL-siderophore
EsaR_Pantoea_stewartii
BAO36750.1_putative_quorum-sensing_LuxR-family_transcriptional_regulator_Serratia_marcescens_SM39
KLX19829.1_hypothetical_protein_SK68_00902_Serratia_marcescens
YenR_Yersinia_enterocolitica
CRY56508.1_quorum-sensing_transcriptional_regulator_Yersinia_intermedia
ESN64571.1_hypothetical_protein_Ser39006_01979_Serratia_sp._ATCC_39006
KOC89253.1_LuxR_family_transcriptional_regulator_Erwinia_iniecta
ABA53179.1_autoinducer-binding_transcriptional_regulator_LuxR_family_Burkholderia_pseudomallei_1710b
ABC34352.1_autoinducer-binding_transcriptional_regulator_LuxR_family_Burkholderia_thailandensis_E264
AKM45289.1_LuxR_family_transcriptional_regulator_Burkholderia_contaminans
RpaR_Rhodopseudomonas_palustris_ATCC_BAA-98
AJQ97082.1_DNA-binding_HTH_domain-containing_protein_Gynuella_sunshinyii_YC6258
AJR05689.1_putative_LuxR_family_transcriptional_regulator_Photobacterium_gaetbulicola_Gung47
ACY15603.1_transcriptional_regulator_LuxR_family_Haliangium_ochraceum_DSM_14365 Lanthipeptide
BjaR_Bradyrhizobium_diazoefficiens_JCM_10833
AFK57307.1_LuxR_family_transcriptional_regulator_(plasmid)_Tistrella_mobilis_KA081020-065
EKF57215.1_putative_transcriptional_regulatory_protein_Agrobacterium_albertimagni_AOL15 AHL-other
KPF41774.1_hypothetical_protein_IP76_19730_Rhizobium_sp._AAP43 AHL-other
ABQ27277.1_transcriptional_regulator_LuxR_family_Geobacter_uraniireducens_Rf4
AGF77635.1_HTH_domain-containing_DNA-binding_protein_Desulfocapsa_sulfexigens_DSM_10523 AHL-ectoine
GAK33572.1_transcriptional_activator_protein_SolR_alpha_proteobacterium_Q-1 Bacteriocin
ERG42748.1_LuxR_family_transcriptional_regulator_Pseudoalteromonas_rubra_DSM_6842
KQQ86281.1_hypothetical_protein_ASF65_05060_Aureimonas_sp._Leaf324
NesR
OryR
KPW40335.1_Transcriptional_regulator_LuxR_family_autoinducer-regulated_Pseudomonas_syringae_pv._apii
KDO00004.1_bacterial_regulatory_s_luxR_family_protein_Pseudomonas_sp._P482 Other
KNX78031.1_LuxR_family_transcriptional_regulator_Pseudomonas_sp._250J
KRQ89860.1_transcriptional_regulator_Bradyrhizobium_valentinum phosphonate
KRR26065.1_transcriptional_regulator_Bradyrhizobium_lablabi
BAM86930.1_conserved_hypothetical_protein_Bradyrhizobium_oligotrophicum_S58
ESX98414.1_LuxR_family_transcriptional_regulator_Mesorhizobium_sp._LNJC399B00
ESZ12173.1_LuxR_family_transcriptional_regulator_Mesorhizobium_sp._L48C026A00
BraR_BtAi1_ABC34629.1_transcriptional_regulator_LuxR_family_Burkholderia_thailandensis_E264
ACA93427.1_transcriptional_regulator_LuxR_family_Burkholderia_cenocepacia_MC0-3
AIO44742.1_bacterial_regulatory_s_luxR_family_protein_Burkholderia_cepacia
ACM38800.1_DNA-binding_HTH_domain-containing_protein_Agrobacterium_vitis_S4
AGT09207.1_transcriptional_regulator_LuxR_family_Paracoccus_aminophilus_JCM_7686
EDQ34151.1_DNA-binding_HTH_domain-containing_protein_Hoeflea_phototrophica_DFL-43 Other
KEC77858.1_LuxR_family_transcriptional_regulator_Sinorhizobium_americanum_CCGM7 Thiopeptide
CAP56781.1_putative_transcriptional_regulator_luxR_family_Gluconacetobacter_diazotrophicus_PA1_5
ACM39362.1_two-component_transcriptional_regulator_LuxR_family_(plasmid)_Agrobacterium_vitis_S4
AHL34515.1_LuxR_family_transcriptional_regulator_Pseudomonas_brassicacearum Bacteriocin
LuxR_Vibrio_fischeri_ES114
ALI08324.1_hypothetical_protein_AO356_16370_Pseudomonas_fluorescens Thiopeptide
LasR_Pseudomonas_aeruginosa_PAO1
ABE35341.1_transcriptional_regulator_LuxR_family_Paraburkholderia_xenovorans_LB400 AHL-bacteriocin
KGM28942.1_LuxR_family_transcriptional_regulator_Photorhabdus_luminescens NRPS-thiopeptide
AAK28504.1_MupR_Pseudomonas_fluorescens
KQV55065.1_hypothetical_protein_ASD07_28510_Duganella_sp._Root336D2 AHL-lanthipeptide
AIO64891.1_bacterial_regulatory_s_luxR_family_protein_Burkholderia_oklahomensis Phosphonate
KGR94889.1_bacterial_regulatory_s_luxR_family_protein_Burkholderia_sp._ABCPW_111 Phosphonate
AJY23787.1_bacterial_regulatory_s_luxR_family_protein_Burkholderia_ambifaria_AMMD Bacteriocin-thiopeptide-phosphonate
KQV49734.1_hypothetical_protein_ASC93_12825_Massilia_sp._Root335 Thiopeptide-PKS
EFI53052.1_transcriptional_regulator_LuxR_family_Afipia_sp._1NLS2 AHL-bacteriocin
AGK57157.1_autoinducer-binding_domain-containing_protein_Hyphomicrobium_denitrificans_1NES1 AHL-bacteriocin
CDX53788.1_Autoinducer-binding_domain_protein_Mesorhizobium_plurifarium AHL-phosphonate
CDX12323.1_LuxR_family_transcriptional_regulator_Mesorhizobium_plurifarium AHL-phosphonate
ABQ36178.1_putative_transcriptional_activator_Bradyrhizobium_sp._BTAi1
CDZ34215.1_Transcriptional_activator_protein_TraR_Neorhizobium_galegae_bv._officinalis AHL-thiopeptide
KQV39924.1_hypothetical_protein_ASC86_22010_Rhizobium_sp._Root1212 AHL-bacteriocin
CDZ34221.1_Quorum-sensing_transcriptional_activator_YpeR_Neorhizobium_galegae_bv._officinalis AHL-thiopeptide
TraR_Agrobacterium_tumefaciens
ESY50944.1_hypothetical_protein_X744_31820_Mesorhizobium_sp._LNJC372A00
KLX13910.1_LuxR_family_transcriptional_regulatory_chaperone_HchA-associated_Serratia_marcescens
KRP45435.1_LuxR_family_transcriptional_regulator_Pseudomonas_libanensis Thiopeptide
KRI25865.1_LuxR_family_transcriptional_regulator_Haematobacter_missouriensis
KNX42195.1_Transcriptional_activator_protein_LasR_Roseovarius_tolerans AHL-terpene
ESX79398.1_LuxR_family_transcriptional_regulator_Mesorhizobium_sp._LSHC414A00 AHL-lanthipeptide
CTQ33376.1_HTH-type_quorum_sensing-dependent_transcriptional_regulator_VjbR_Jannaschia_rubra
KAJ55423.1_hypothetical_protein_ACMU_12055_Actibacterium_mucosum_KCTC_23349
KEO60115.1_hypothetical_protein_DT23_14585_Thioclava_indica AHL-bacteriocin
KPN63446.1_LuxR_family_transcriptional_regulator_Aliiroseovarius_crassostreae AHL-lanthipeptide
ETW11960.1_LuxR_family_transcriptional_regulator_Roseivivax_atlanticus Ectoine
KFL90810.1_Transcriptional_activator_protein_cwiR_Acetobacter_malorum AHL-terpene
ABD87583.1_transcriptional_regulator_LuxR_family_Rhodopseudomonas_palustris_BisB18
ABC94329.1_transcriptional_regulator_(activator)_protein_LuxR_family_(plasmid)_Rhizobium_etli_CFN_42
EJC75477.1_response_regulator_containing_a_CheY-like_receiver_domain_and_an_HTH_DNA-binding_domain_Rhizobium_leguminosarum_bv._trifolii_WSM2012
AHF88043.1_LuxR_family_transcriptional_regulator_(plasmid)_Rhizobium_leguminosarum_bv._trifolii_WSM1689 AHL-bacteriocin
BAI74134.1_transcriptional_regulator_(plasmid)_Azospirillum_sp._B510
KQZ48532.1_DNA-binding_protein_Rhizobium_sp._Root149 Thiopeptide
AEA60277.1_Transcriptional_regulator_LuxR_family_protein_Burkholderia_gladioli_BSR3 Bacteriocin-NRPS
AJK45852.1_transcriptional_regulator_LuxR_family_Burkholderia_glumae_PG1 Bacteriocin
CDN63731.1_putative_transcriptional_regulator_of_trehalose_utilization_LuxR_family_Burkholderia_cenocepacia_H111 Terpene
AKM38762.1_LuxR_family_transcriptional_regulator_Burkholderia_contaminans Terpene
AJK50818.1_putative_transcriptional_regulator_LuxR_family_Burkholderia_glumae_PG1 Betalactam
AKQ13286.1_putative_LuxR_family_transcription_regulator_Streptomyces_canus
AFO93570.1_putative_HTH-type_transcriptional_regulator_(plasmid)_Phaeobacter_inhibens_DSM_17395 Siderophore
GerE
0.05
