# Supplementary material for: luxR Homolog-Linked Biosynthetic Gene Clusters in Proteobacteria
Source: mSystems. 2018 Mar 27;3(3):e00208-17. doi: 10.1128/mSystems.00208-17 (PMC5872303; doi:10.1128/mSystems.00208-17)
Supplement: DATA SET S1 [file sys003182212sd1.pdf]

Organism	Nucleotide accession	Complete genome	BGC type	Protein accession LuxR hit	Annotation	Locus tag LuxR hit	LuxR hit coordinates	LuxI hit?	LuxI hit accession(s)	Class	Order	Family	Genus	Lifestyle	Characterized Product?
Acetobacter malorum strain DmCS_005 contig56, whole genome shotgun sequence.	JOJU01000026	NO	AHL-terpene	KFL90810	Transcriptional_activator_protein_raiR	AmDm5_0758	31375-32115	Yes	KFL90811	Alphaproteobacteria	Rhodospirillales	Acetobacteraceae	Acetobacter	Other (Insect symbiont)	
Achromobacter sp. Root83 contig_7, whole genome shotgun sequence.	LMIU01000026	NO	AHL-NRPS-PKS	KRC69210	LuxR_family_transcriptional_regulator	ASE30_18440	52950-53663	Yes	KRC69211	Betaproteobacteria	Burkholderiales	Alcaligenaceae	Achromobacter	Plant associated	
Achromobacter xylosoxidans A8, complete genome.	CP002287	YES	NRPS	ADP15908	bacterial_regulatory_protein,_LuxR_family	AXYL_02587	2748935-2749648	No		Betaproteobacteria	Burkholderiales	Alcaligenaceae	Achromobacter	Environmental	
Acidovorax sp. SD340 contig_8, whole genome shotgun sequence.	LHUP01000133	NO	AHL-NRPS-PKS	KQB59097	LuxR_family_transcriptional_regulator	AE621_11680	17631-18329	Yes	KQB59096	Betaproteobacteria	Burkholderiales	Comamonadaceae	Acidovorax	Environmental	
Acinetobacter baumannii str. AYE, complete genome.	CU459141	YES	AHL-NRPS	CAM88520	putative_Autoinducer-binding_transcriptional	ABAYE3758	3793249-3793965	Yes	CAM88522	Gammaproteobacteria	Pseudomonadales	Moraxellaceae	Acinetobacter	Human associated	
Acinetobacter calcoaceticus NIPH 13 acLrG-supercont1.3.C5, whole genome shotgun sequence.	APOE01000005	NO	AHL-NRPS	ENU09936	hypothetical_protein	F997_00969	38979-39695	Yes	ENU09934	Gammaproteobacteria	Pseudomonadales	Moraxellaceae	Acinetobacter	Human associated	
Acinetobacter pittii ANC 4052 acMaa-supercont1.4.C4, whole genome shotgun sequence.	APQO01000004	NO	AHL-NRPS	EOQ74914	hypothetical_protein	F929_01033	689479-690195	Yes	EOQ74912	Gammaproteobacteria	Pseudomonadales	Moraxellaceae	Acinetobacter	Human associated	
Actibacterium mucosum KCTC 23349 contig4, whole genome shotgun sequence.	JFKE01000004	NO	AHL-PKS	KAJ55423	hypothetical_protein	ACMU_12055	198138-198749	Yes	KAJ55422	Alphaproteobacteria	Rhodobacterales	Rhodobacteraceae	Actibacterium	Environmental	
Afipia sp. 1NLS2 ctg00009, whole genome shotgun sequence.	ADVZ01000001	NO	AHL-bacteriocin	EFI53052	transcriptional_regulator,_LuxR_family	AfiDRAFT_1039	1060712-1061437	Yes	EFI53051	Alphaproteobacteria	Rhizobiales	Bradyrhizobiaceae	Afipia	Environmental	
Agrobacterium albertimagni AOL15 Contig_23, whole genome shotgun sequence.	ALJF01000023	NO	AHL-other	EKF57215	putative_transcriptional_regulatory_protein	QWE_22786	186007-186747	Yes	EKF57214	Alphaproteobacteria	Rhizobiales	Rhizobiaceae	Agrobacterium	Environmental	
Agrobacterium vitis S4 chromosome 2, complete sequence.	CP000634	YES	NRPS	ACM38800	DNA-binding_HTH_domain-containing_protein	Avi_5725	746023-746946	No		Alphaproteobacteria	Rhizobiales	Rhizobiaceae	Agrobacterium	Plant associated	
Agrobacterium vitis S4 plasmid pAtS4b, complete sequence.	CP000635	YES	AHL-NRPS	ACM39362	two-component_transcriptional_regulator_LuxR	Avi_9665	128401-129123	Yes	ACM39363	Alphaproteobacteria	Rhizobiales	Rhizobiaceae	Agrobacterium	Plant associated	
Aliiroseovarius crassostreae strain CV919-312 Joined_contig_7, whole genome shotgun sequence.	LKBA01000006	NO	AHL-lanthipeptide	KPN63446	LuxR_family_transcriptional_regulator	AKJ29_12385	414519-415130	Yes	KPN63447	Alphaproteobacteria	Rhodobacterales	Rhodobacteraceae	Aliiroseovarius	Other (Oyster pathogen)	
Alpha proteobacterium Q-1 DNA, contig: contig000015, whole genome shotgun sequence.	BAYV01000015	NO	bacteriocin	GAK33572	transcriptional_activator_protein_SolR	AQ1_01462	25427-26125	No		Alphaproteobacteria				Environmental	
Aureimonas sp. Leaf324 contig_2, whole genome shotgun sequence.	LMOH01000012	NO	NRPS-PKS	KQQ86281	hypothetical_protein	ASF65_05060	413013-413765	No		Alphaproteobacteria	Rhizobiales	Aurantimonadaceae	Aureimonas	Plant associated	
Azospirillum sp. B510 plasmid pAB510a DNA, complete genome.	AP010947	YES	AHL-NRPS-PKS	BAI74134	transcriptional_regulator	AZL_a06030	768921-769784	Yes	BAI74120	Alphaproteobacteria	Rhodospirillales	Rhodospirillaceae	Azospirillum	Plant associated	
Bradyrhizobium lablabi strain CCBAU 23086 scaffold18, whole genome shotgun sequence.	LLYB01000050	NO	NRPS	KRR26065	transcriptional_regulator	CQ14_38350	3554-4276	No		Alphaproteobacteria	Rhizobiales	Bradyrhizobiaceae	Bradyrhizobium	Plant associated	
Bradyrhizobium oligotrophicum S58 DNA, complete genome.	AP012603	YES	AHL-NRPS	BAM86930	conserved_hypothetical_protein	S58_09190	1027505-1028296	Yes	BAM86929	Alphaproteobacteria	Rhizobiales	Bradyrhizobiaceae	Bradyrhizobium	Plant associated	
Bradyrhizobium sp. BTAi1, complete genome.	CP000494	YES	NRPS	ABQ36178	putative_transcriptional_activator	BBta_4114	4321652-4322293	No		Alphaproteobacteria	Rhizobiales	Bradyrhizobiaceae	Bradyrhizobium	Plant associated	
Bradyrhizobium valentinum strain LmjM6 contig_94, whole genome shotgun sequence.	LLXY01000237	NO	phosphonate	KRQ89860	transcriptional_regulator	CQ10_37630	15402-16163	No		Alphaproteobacteria	Rhizobiales	Bradyrhizobiaceae	Bradyrhizobium	Plant associated	
Burkholderia ambifaria AMMD chromosome 2, complete sequence.	CP009799	YES	iocin-Thiopeptide-Phos	AJY23787	bacterial_regulatory_s,_luxR_family_protein	CH72_5081	2043811-2044587	No		Betaproteobacteria	Burkholderiales	Burkholderiaceae	Burkholderia	Human associated	
Burkholderia ambifaria AMMD chromosome 3, complete sequence.	CP000442	YES	PKS	ABI91455	transcriptional_regulator,_LuxR_family	Bamb_5910	393560-394273	No		Betaproteobacteria	Burkholderiales	Burkholderiaceae	Burkholderia	Human associated	enacyloxin
Burkholderia ambifaria MC40-6 chromosome 3, complete sequence.	CP001027	YES	Other	ACB68142	transcriptional_regulator,_LuxR_family	BamMC406_5699	211136-211855	No		Betaproteobacteria	Burkholderiales	Burkholderiaceae	Burkholderia	Human associated	
Burkholderia cenocepacia H111 chromosome 2, complete genome.	HG938371	YES	terpene	CDN63731	utative_transcriptional_regulator_of_trehalo	I35_5895	2139385-2140398	No		Betaproteobacteria	Burkholderiales	Burkholderiaceae	Burkholderia	Human associated	
Burkholderia cenocepacia MC0-3 chromosome 2, complete sequence.	CP000959	YES	AHL-NRPS	ACA93445	transcriptional_regulator,_LuxR_family	Bcenmc03_4299	1256387-1257100	Yes	ACA93428	Betaproteobacteria	Burkholderiales	Burkholderiaceae	Burkholderia	Plant associated	
Burkholderia cenocepacia MC0-3 chromosome 2, complete sequence.	CP000959	YES	AHL-NRPS	ACA93427	transcriptional_regulator,_LuxR_family	Bcenmc03_4279	1233950-1234915	Yes	ACA93428	Betaproteobacteria	Burkholderiales	Burkholderiaceae	Burkholderia	Plant associated	
Burkholderia cepacia strain DDS 7H-2 chromosome 2, complete sequence.	CP007786	YES	NRPS	AIO44742	bacterial_regulatory_s,_luxR_family_protein	DM42_4942	1564305-1565348	No		Betaproteobacteria	Burkholderiales	Burkholderiaceae	Burkholderia	Environmental	
Burkholderia contaminans strain MS14 chromosome 1, complete sequence.	CP009743	YES	Terpene	AKM38762	LuxR_family_transcriptional_regulator	NL30_02000	472153-473001	No		Betaproteobacteria	Burkholderiales	Burkholderiaceae	Burkholderia	Environmental	
Burkholderia contaminans strain MS14 chromosome 3, complete sequence.	CP009745	YES	PKS	AKM45289	LuxR_family_transcriptional_regulator	NL30_36195	1254782-1255495	No		Betaproteobacteria	Burkholderiales	Burkholderiaceae	Burkholderia	Environmental	
Burkholderia gladioli BSR3 chromosome 1, complete sequence.	CP002599	YES	Bacteriocin-NRPS	AEA60277	ranscriptional_regulator,_LuxR_family_protei	bgla_1g16270	1809175-1810056	No		Betaproteobacteria	Burkholderiales	Burkholderiaceae	Burkholderia	Plant associated	
Burkholderia gladioli BSR3 chromosome 2, complete sequence.	CP002600	YES	AHL-Other	AEA63556	quorum_sensing_LuxR_family_sensor_regulator	bgla_2g11070	1395508-1396230	Yes	AEA63554	Betaproteobacteria	Burkholderiales	Burkholderiaceae	Burkholderia	Plant associated	
Burkholderia gladioli BSR3 plasmid bgla_1p, complete sequence.	CP002601	YES	NRPS-PKS	AEA65567	transcriptional_regulator,_LuxR_family	bgla_1p1760	237847-238560	Yes	AEA65565	Betaproteobacteria	Burkholderiales	Burkholderiaceae	Burkholderia	Plant associated	
Burkholderia glumae BGR1 chromosome 1, complete sequence.	CP001503	YES	NRPS-PKS	ACR28842	utoinducer-binding_transcriptional_regulato	bglu_1g17030	1889718-1890422	No		Betaproteobacteria	Burkholderiales	Burkholderiaceae	Burkholderia	Plant associated	
Burkholderia glumae PG1 chromosome 1, complete sequence.	CP002580	YES	Bacteriocin	AJK45952	transcriptional_regulator,_LuxR_family	BGL_1c14360	1666527-1667270	No		Betaproteobacteria	Burkholderiales	Burkholderiaceae	Burkholderia	Plant associated	
Burkholderia glumae PG1 chromosome 1, complete sequence.	CP002580	YES	NRPS	AJK46550	autoinducer-binding_transcriptionalregulator	BGL_1c20410	2335060-2335773	No		Betaproteobacteria	Burkholderiales	Burkholderiaceae	Burkholderia	Plant associated	
Burkholderia glumae PG1 chromosome 2, complete sequence.	CP002581	YES	AHL-NRPS	AJK48490	transcriptional_activator_protein_LasR	BGL_2c03990	487465-488172	Yes	AJK48489	Betaproteobacteria	Burkholderiales	Burkholderiaceae	Burkholderia	Plant associated	
Burkholderia glumae PG1 chromosome 2, complete sequence.	CP002581	YES	Betalactam	AJK50818	utative_transcriptional_regulator,_LuxR_fami	BGL_2c27640	3478105-3478818	No		Betaproteobacteria	Burkholderiales	Burkholderiaceae	Burkholderia	Plant associated	
Burkholderia glumae PG1 chromosome 2, complete sequence.	CP002581	YES	NRPS	AJK49718	transcriptional_regulator,_LuxR_family	BGL_2c16510	2068251-2068964	No		Betaproteobacteria	Burkholderiales	Burkholderiaceae	Burkholderia	Plant associated	
Burkholderia oklahomensis strain EO147 chromosome 1, complete sequence.	CP008726	YES	phosphonate	AIO64891	bacterial_regulatory_s,_luxR_family_protein	DM82_2524	2891358-2892089	No		Betaproteobacteria	Burkholderiales	Burkholderiaceae	Burkholderia	Human associated	
Burkholderia pseudomallei 1710b chromosome II, complete sequence.	CP000125	YES	PKS	ABA53179	utoinducer-binding_transcriptional_regulato	BURPS1710b_A1863	2276711-2277895	No		Betaproteobacteria	Burkholderiales	Burkholderiaceae	Burkholderia	Human associated	
Burkholderia pseudomallei ABCPW 111 X946.contig-100_7, whole genome shotgun sequence.	JPWT01000110	NO	phosphonate	KGR94889	bacterial_regulatory_s,_luxR_family_protein	X946_1511	24888-25619	No		Betaproteobacteria	Burkholderiales	Burkholderiaceae	Burkholderia	Environmental	
Burkholderia pyrrocinia strain Lyc2 contig_9, whole genome shotgun sequence.	JPWP01000009	NO	NRPS-PKS	KFL51902	LuxR_family_transcriptional_regulator	JM78_19380	67667-68386	No		Betaproteobacteria	Burkholderiales	Burkholderiaceae	Burkholderia	Plant associated	
Burkholderia thailandensis E264 chromosome II, complete sequence.	CP000085	YES	NRPS-PKS-Thiopeptide	ABC34138	utoinducer-binding_transcriptional_regulato	BTH_II1681	2036259-2036975	No		Betaproteobacteria	Burkholderiales	Burkholderiaceae	Burkholderia	Environmental	thailandamide
Burkholderia thailandensis E264 chromosome II, complete sequence.	CP000085	YES	PKS	ABC34352	utoinducer-binding_transcriptional_regulato	BTH_II2087	2543245-2544522	No		Betaproteobacteria	Burkholderiales	Burkholderiaceae	Burkholderia	Environmental	malleilactone
Burkholderia thailandensis E264 chromosome II, complete sequence.	CP000085	YES	AHL-NRPS-PKS	ABC34774	utoinducer-binding_transcriptional_regulato	BTH_II1231	1454107-1454811	Yes	ABC34067	Betaproteobacteria	Burkholderiales	Burkholderiaceae	Burkholderia	Environmental	bactobolin
Burkholderia thailandensis H0587 chromosome 2, complete sequence.	CP004090	YES	AHL-NRPS	AHI66402	bacterial_regulatory_s,_luxR_family_protein	BTL_4170	976296-977009	Yes	AHI67412	Betaproteobacteria	Burkholderiales	Burkholderiaceae	Burkholderia	Human associated	
Burkholderia ubonensis MSMB22 chromosome II, complete sequence.	CP009486	YES	AHL-Terpene	AJX12009	sigma-70,_region_4_family_protein	BW23_5523	2564432-2565151	Yes	AJX12200	Betaproteobacteria	Burkholderiales	Burkholderiaceae	Burkholderia	Human associated	
Burkholderia ubonensis MSMB22 chromosome III, complete sequence.	CP009487	YES	NRPS-PKS-Bacteriocin	AJX14779	bacterial_regulatory_s,_luxR_family_protein	BW23_5801	239048-239827	No		Betaproteobacteria	Burkholderiales	Burkholderiaceae	Burkholderia	?	
Burkholderia xenovorans LB400 chromosome 2, complete sequence.	CP000271	YES	AHL-bacteriocin	ABE35341	transcriptional_regulator,_LuxR_family	Bxe_B0610	2694331-2695035	Yes	ABE35343	Betaproteobacteria	Burkholderiales	Burkholderiaceae	Burkholderia	Environmental	
Citrobacter freundii strain MRSN 12115 scaffold00001, whole genome shotgun sequence.	JYFZ02000001	NO	Other	KJC09793	hypothetical_protein	TO64_04970	1756253-1756870	No		Gammaproteobacteria	Enterobacterales	Enterobacteriaceae	Citrobacter	Human associated	
Desulfocapsa sulfexigens DSM 10523, complete genome.	CP003985	YES	AHL-Ectoine	AGF77635	HTH_domain-containing_DNA-binding_protein	UWK_01063	1204412-1205143	Yes	AGF77637	Deltaproteobacteria	Desulfobacterales	Desulfobulbaceae	Desulfocapsa	Environmental	
Duganella sp. Leaf61 contig_18, whole genome shotgun sequence.	LMLM01000010	NO	AHL-NRPS	KQN76037	LuxR_family_transcriptional_regulator	ASF04_25895	1783-2505	Yes	KQN76038, KQN76055	Betaproteobacteria	Burkholderiales	Oxalobacteraceae	Duganella	Plant associated	
Duganella sp. Root336D2 contig_17, whole genome shotgun sequence.	LMDB01000009	NO	AHL-lanthipeptide	KQV55085	hypothetical_protein	ASD07_28510	45006-45620	Yes	KQV55061, KQV55062	Betaproteobacteria	Burkholderiales	Oxalobacteraceae	Duganella	Plant associated	
Erwinia carotovora	U17224	YES	Betalactam	AAC45995	CarR	no_locus_tag	691-1425	No		Gammaproteobacteria	Enterobacterales	Pectobacteriaceae	Pectobacterium	Plant associated	1-carbapen-2-em 3-carboxylic acid
Erwinia iniecta strain B120 contig000017, whole genome shotgun sequence.	JRXE01000017	NO	NRPS	KOC89253	LuxR_family_transcriptional_regulator	NG42_13050	27855-28598	No		Gammaproteobacteria	Enterobacterales	Pectobacteriaceae	Pectobacterium	Other (Insect symbiont)	
Escherichia coli KTE67 acEvz-supercont1.2.C21, whole genome shotgun sequence.	ANUM01000021	NO	NRPS-PKS	ELE40727	hypothetical_protein	A1U7_02824	377356-377970	No		Gammaproteobacteria	Enterobacterales	Enterobacteriaceae	Escherichia	Human associated	
Frateuria aurantia DSM 6220, complete genome.	CP003350	YES	terpene	AFC85684	DNA-binding_protein_with_HTH_domain	Fraau_1240	1338209-1338940	No		Gammaproteobacteria	Xanthomonadales	Rhodanobacteraceae	Frateuria	Plant associated	
Geobacter uraniireducens Rf4, complete genome.	CP000698	YES	AHL-PKS	ABQ27277	transcriptional_regulator,_LuxR_family	Gura_3112	3680391-3681143	Yes	ABQ27276	Deltaproteobacteria	Desulfuromonadales	Geobacteraceae	Geobacter	Environmental	
Gluconacetobacter diazotrophicus PAl 5 complete genome.	AM889285	YES	AHL-NRPS	CAP56781	utative_transcriptional_regulator,_luxR_fami	GDI2838	2930022-2930741	Yes	CAP56779	Alphaproteobacteria	Rhodospirillales	Acetobacteraceae	Gluconacetobacter	Plant associated	
Gynuella sunshinyii YC6258, complete genome.	CP007142	YES	AHL-NRPS-PKS	AJQ97082	DNA-binding_HTH_domain-containing_protein	YC6258_05052	5438075-5438818	Yes	AJQ97077	Gammaproteobacteria	Oceanospirillales	Saccharospirillaceae	Gynuella	Environmental	
Haematobacter missouriensis strain CCUG 52307 contig39_scaffold29, whole genome shotgun sequence.	JFGS01000035	NO	NRPS	KFI25865	LuxR_family_transcriptional_regulator	CG51_12825	12263-12982	No		Alphaproteobacteria	Rhodobacterales	Rhodobacteraceae	Haematobacter	Human associated	
Haliangium ochraceum DSM 14365, complete genome.	CP001804	YES	Lanthipeptide	ACY15603	transcriptional_regulator,_LuxR_family	Hoch_3098	4269243-4270007	No		Deltaproteobacteria	Myxococcales	Nannocystineae	Kolferiaceae	Environmental	
Halothiobacillus neapolitanus c2, complete genome.	CP001801	YES	AHL-PKS	ACX95801	transcriptional_regulator,_LuxR_family	Hneap_0963	1039450-1040163	Yes	ACX95803	Gammaproteobacteria	Chromatiales	Halothiobacillaceae	Halothiobacillus	Environmental	
Hoeflea phototrophica DFL-43 HPDFL_4, whole genome shotgun sequence.	ABIA03000004	NO	Other	EDQ34151	DNA-binding_HTH_domain-containing_protein	HPDFL43_14177	643357-644070	No		Alphaproteobacteria	Rhizobiales	Phyllobacteriaceae	Hoeflea	Environmental	
Hoeflea sp. IMCC20628, complete genome.	CP011479	YES	PKS	AKI01950	DNA-binding_protein_with_HTH_domain	IMCC20628_03257	3412382-3413086	No		Alphaproteobacteria	Rhizobiales	Phyllobacteriaceae	Hoeflea	Environmental	
Hyphomicrobium denitrificans 1NES1, complete genome.	CP005587	YES	AHL-bacteriocin	AGK57157	autoinducer-binding_domain-containing_protei	HYPDE_27388	950229-951074	Yes	AGK57158	Alphaproteobacteria	Rhizobiales	Hyphomicrobiaceae	Hyphomicrobium	Environmental	
Jannaschia rubra genome assembly J.rubraCECT5088_SeqMan_Prokka, contig 0020, whole genome shotgun sequence.	CXPG01000020	NO	AHL-PKS	CTQ33376	HTH-type_quorum_sensing-dependent	JAN5088_02158	208319-209029	Yes	CTQ33377	Alphaproteobacteria	Rhodobacterales	Rhodobacteraceae	Jannaschia	Environmental	
Massilia sp. Root335 contig_21, whole genome shotgun sequence.	LMCY01000011	NO	Thiopeptide-PKS	KQV49734	hypothetical_protein	ASC93_12825	259639-260328	No		Betaproteobacteria	Burkholderiales	Oxalobacteraceae	Massilia	Plant associated	
n plurifarium genome assembly Mesorhizobium plurifarium ORS1032T genome assembly, contig MPL1032_Contig_18, whole genome shot	CCND01000010	NO	AHL-phosphonate	CDX53788	Autoinducer-binding_domain_protein	MPL1032_180155	132986-133711	Yes	CDX53842	Alphaproteobacteria	Rhizobiales	Phyllobacteriaceae	Mesorhizobium	Plant associated	
Mesorhizobium plurifarium genome assembly ORS3356-Illumina, contig MPL3356_Contig_11, whole genome shotgun sequence.	CCMZ01000003	NO	AHL-phosphonate	CDX12323	LuxR_family_transcriptional_regulator	MPL3356_110444	420138-420866	Yes	CDX12320	Alphaproteobacteria	Rhizobiales	Phyllobacteriaceae	Mesorhizobium	Plant associated	
Mesorhizobium sp. L48C026A00 scaffold0022, whole genome shotgun sequence.	AYWU01000022	NO	NRPS-PKS	ESZ12173	LuxR_family_transcriptional_regulator	X737_27605	45637-46494	No		Alphaproteobacteria	Rhizobiales	Phyllobacteriaceae	Mesorhizobium	Plant associated	
Mesorhizobium sp. LNJC372A00 scaffold0026, whole genome shotgun sequence.	AYWN01000026	NO	NRPS-PKS	ESY50944	hypothetical_protein	X744_31820	26802-27539	No		Alphaproteobacteria	Rhizobiales	Phyllobacteriaceae	Mesorhizobium	Plant associated	
Mesorhizobium sp. LNJC399B00 scaffold0014, whole genome shotgun sequence.	AYWE01000014	NO	NRPS	ESX98414	LuxR_family_transcriptional_regulator	X753_31275	22081-22890	No		Alphaproteobacteria	Rhizobiales	Phyllobacteriaceae	Mesorhizobium	Plant associated	
Mesorhizobium sp. LSHC414A00 scaffold0001, whole genome shotgun sequence.	AYWA01000001	NO	AHL-lanthipeptide	ESX79398	LuxR_family_transcriptional_regulator	X757_03125	639059-639838	Yes	ESX79397	Alphaproteobacteria	Rhizobiales	Phyllobacteriaceae	Mesorhizobium	Plant associated	
Methylobacter tundripaludum SV96 genomic scaffold Mettuscaffold_1, whole genome shotgun sequence.	JH109152	YES	AHL-Other	EGW22657	transcriptional_regulator,_LuxR_family	Mettu_1481	1540483-1541193	Yes	EGW22655	Gammaproteobacteria	Methylococcales	Methylococcaceae	Methylobacter	Environmental	*See:* Puri AW, et al. J Bacteriol JB-00773.
Neorhizobium galegae bv. officinalis genome assembly NgalHAMBI1146Draft, contig contig2, whole genome shotgun sequence.	CCRI01000002	NO	AHL-thiopeptide	CDZ34215	Transcriptional_activator_protein_TraR	NGAL_HAMBI1146_07490	47187-47891	Yes	CDZ34213	Alphaproteobacteria	Rhizobiales	Rhizobiaceae	Agrobacterium	Plant associated	
Neorhizobium galegae bv. officinalis genome assembly NgalHAMBI1146Draft, contig contig2, whole genome shotgun sequence.	CCRI01000002	NO	AHL-thiopeptide	CDZ34221	Quorum-sensing_transcriptional_activator_Ype	NGAL_HAMBI1146_07500	47985-48686	Yes	CDZ34213	Alphaproteobacteria	Rhizobiales	Rhizobiaceae	Agrobacterium	Plant associated	
Pantoea vagans C9-1 plasmid pPag3, complete sequence.	CP001895	YES	AHL-terpene	ADI78210	Transcriptional_activator_protein_PagR	Pvag_pPag30141	136873-137565	Yes	ADI78211	Gammaproteobacteria	Enterobacterales	Erwiniaceae	Pantoea	Plant associated	
Paracoccus aminophilus JCM 7686, complete genome.	CP006650	YES	AHL-PKS	AGT09207	transcriptional_regulator,_LuxR_family	JCM7686_2126	2195654-2196430	Yes	AGT09205	Alphaproteobacteria	Rhodobacterales	Rhodobacteraceae	Paracoccus	Environmental	
Pectobacterium carotovorum subsp. odoriferum strain BC S7, complete genome.	CP009678	YES	NRPS-PKS	AIU89025	hypothetical_protein	BCS7_13615	3092448-3093137	No		Gammaproteobacteria	Enterobacterales	Pectobacteriaceae	Pectobacterium	Plant associated	
Phaeobacter inhibens DSM 17395 plasmid pPGA1_78, complete sequence.	CP002978	YES	Siderophore	AFO93570	putative_HTH-type_transcriptional_regulator	PGA1_78p00490	60744-61784	No		Alphaproteobacteria	Rhodobacterales	Rhodobacteraceae	Phaeobacter	Environmental	
Photobacterium gaetbulicola Gung47 chromosome 1, complete sequence.	CP005973	YES	NRPS-PKS	AJR05689	utative_LuxR_family_transcriptional_regulato	H744_1c0664	792772-793551	No		Gammaproteobacteria	Vibrionales	Vibrionaceae	Photobacterium	Environmental	
Photorhabdus luminescens strain LN2 Scaffold2, whole genome shotgun sequence.	JQOC01000002	NO	NRPS-Thiopeptide	KGM28942	LuxR_family_transcriptional_regulator	KS18_03945	47799-48494	No		Gammaproteobacteria	Enterobacterales	Morganellaceae	Photorhabdus	Other (Nematode symbiont)	
Photorhabdus temperata subsp. thracensis strain DSM 15199, complete genome.	CP011104	YES	Betalactam	AKH63559	transcriptional_regulator	VY86_09640	2242923-2243651	No		Gammaproteobacteria	Enterobacterales	Morganellaceae	Photorhabdus	Other (Nematode symbiont)	
Pseudoalteromonas citrea DSM 8771 strain NCIMB 1889 contig138, whole genome shotgun sequence.	AHBZ02000138	NO	AHL-PKS	ERG18257	LuxR_family_transcriptional_regulator	PCIT_13149	114685-115431	Yes	ERG18256	Gammaproteobacteria	Alteromonadales	Pseudoalteromonadaceae	Pseudoalteromonas	Environmental	
Pseudoalteromonas rubra DSM 6842 strain ATCC 29570 contig170, whole genome shotgun sequence.	AHCD02000170	NO	NRPS-PKS	ERG42748	LuxR_family_transcriptional_regulator	PRUB_22926	134762-135475	No		Gammaproteobacteria	Alteromonadales	Pseudoalteromonadaceae	Pseudoalteromonas	Environmental	
Pseudomonas aeruginosa PAO1, complete genome.	AE004091	YES	Phenazine	AAG05287	quorum-sensing_control_repressor	PA1898	2069490-2070203	No		Gammaproteobacteria	Pseudomonadales	Pseudomonadaceae	Pseudomonas	Environmental/ human associated	Phenazine
Pseudomonas batumici strain UCM B-321 Contig_127, whole genome shotgun sequence.	JXDG01000126	NO	AHL-Phenazine	KIH80457	Transcriptional_regulator_RhlR	UCMB321_5802	45657-46379	Yes	KIH80458	Gammaproteobacteria	Pseudomonadales	Pseudomonadaceae	Pseudomonas	Plant associated	
Pseudomonas batumici strain UCM B-321 Contig_32, whole genome shotgun sequence.	JXDG01000032	NO	AHL-NRPS	KIH83862	Quorum-sensing_transcriptional_activator_Ype	UCMB321_2444	80453-80929	Yes	KIH83864	Gammaproteobacteria	Pseudomonadales	Pseudomonadaceae	Pseudomonas	Plant associated	
Pseudomonas brassicacearum strain DF41, complete genome.	CP007410	YES	Bacteriocin	AHL34515	LuxR_family_transcriptional_regulator	CD58_17135	3916001-3916795	No		Gammaproteobacteria	Pseudomonadales	Pseudomonadaceae	Pseudomonas	Plant associated	
Pseudomonas chlororaphis	AF195615	YES	AHL-Phenazine	AAF17494	transcriptional_activator_PhzR	no_locus_tag	845-1570	Yes	AAF17493	Gammaproteobacteria	Pseudomonadales	Pseudomonadaceae	Pseudomonas	Plant associated	
Pseudomonas cichorii JBC1, complete genome.	CP007039	YES	NRPS	AHF67689	hypothetical_protein	PCH70_25360	2962759-2963490	No		Gammaproteobacteria	Pseudomonadales	Pseudomonadaceae	Pseudomonas	Plant associated	
Pseudomonas corrugata strain CFBP 5454	KF192265	YES	AHL-NRPS	ABP88722	transcriptional_activator_protein	no_locus_tag	486-1214	Yes	ABP88724	Gammaproteobacteria	Pseudomonadales	Pseudomonadaceae	Pseudomonas	Plant associated	Corrugatin
Pseudomonas extremaustralis 14-3 substr. 14-3b strain 14-3 contig00001, whole genome shotgun sequence.	AHIP01000001	NO	Other	EZI30475	LuxR_family_transcriptional_regulator	PE143B_0101075	236932-237642	No		Gammaproteobacteria	Pseudomonadales	Pseudomonadaceae	Pseudomonas	Environmental	
Pseudomonas fluorescens autoinducer synthase (phzI) gene, positive regulator protein (phzR), phzABCDEFG genes, complete cds.	L48616	YES	AHL-Phenazine	AAC18899		no_locus_tag	717-1451	Yes	AAC18898	Gammaproteobacteria	Pseudomonadales	Pseudomonadaceae	Pseudomonas	Plant associated	
Pseudomonas fluorescens FH5 scf_29078_59.1_contig_1, whole genome shotgun sequence.	AOJA01000070	NO	NRPS	ETK39104	hypothetical_protein	H098_23930	59987-60625	No		Gammaproteobacteria	Pseudomonadales	Pseudomonadaceae	Pseudomonas	Environmental	
Pseudomonas fluorescens NCIMB 10586 mupirocin biosynthetic gene cluster.	AF318063	YES	AHL-PKS	AAK28504	MupR	no_locus_tag	71638-72342	Yes	AAK28505	Gammaproteobacteria	Pseudomonadales	Pseudomonadaceae	Pseudomonas	Plant associated	Mupirocin
Pseudomonas fluorescens Q2-87 contig00001, whole genome shotgun sequence.	AGBM01000001	NO	Lanthipeptide	EJL01815	utoinducer-binding_transcriptional_regulato	PflQ2_4761	851832-852545	No		Gammaproteobacteria	Pseudomonadales	Pseudomonadaceae	Pseudomonas	Plant associated	
Pseudomonas fluorescens strain C2 contig_13, whole genome shotgun sequence.	JSAK01000013	NO	Lanthipeptide	KII32728	LuxR_family_transcriptional_regulator	NL64_11455	127257-127988	No		Gammaproteobacteria	Pseudomonadales	Pseudomonadaceae	Pseudomonas	Plant associated	
Pseudomonas fluorescens strain FW300-N2C3, complete genome.	CP012831	YES	AHL-NRPS	ALI10528	hypothetical_protein	AO356_28160	6433429-6434157	Yes	ALI10530	Gammaproteobacteria	Pseudomonadales	Pseudomonadaceae	Pseudomonas	Environmental	
Pseudomonas fluorescens strain FW300-N2C3, complete genome.	CP012831	YES	Thiopeptide	ALI08324	hypothetical_protein	AO356_16370	3699364-3700089	No		Gammaproteobacteria	Pseudomonadales	Pseudomonadaceae	Pseudomonas	Environmental	
Pseudomonas libanensis strain DSM 17149 7_326504_15.2974, whole genome shotgun sequence.	JYLH01000007	NO	Thiopeptide	KRP45435	LuxR_family_transcriptional_regulator	TU73_13630	243738-244457	No		Gammaproteobacteria	Pseudomonadales	Pseudomonadaceae	Pseudomonas	Environmental	
Pseudomonas libanensis strain DSM 17149 8_257449_21.4035, whole genome shotgun sequence.	JYLH01000008	NO	Phenazine	KRP45081	transcriptional_regulator	TU73_14400	94279-95004	No		Gammaproteobacteria	Pseudomonadales	Pseudomonadaceae	Pseudomonas	Environmental	
Pseudomonas sp. 250J 250J_85, whole genome shotgun sequence.	JHEE01000085	NO	NRPS-PKS	KNX78031	LuxR_family_transcriptional_regulator	DA83_25100	62513-63274	No		Gammaproteobacteria	Pseudomonadales	Pseudomonadaceae	Pseudomonas	Environmental	
Pseudomonas sp. CMAA1215 Contig96, whole genome shotgun sequence.	AVOY01000221	NO	AHL-phenazine-PKS	ERO59965	transcriptional_regulator	P308_15775	1703-2227	Yes	ERO59966	Gammaproteobacteria	Pseudomonadales	Pseudomonadaceae	Pseudomonas	Plant associated	
Pseudomonas sp. DSM 29164 11_178365_15.817, whole genome shotgun sequence.	JYLN01000011	NO	Thiopeptide	KRP69514	transcriptional_regulator	TX23_23370	83218-83772	No		Gammaproteobacteria	Pseudomonadales	Pseudomonadaceae	Pseudomonas	Environmental	
Pseudomonas sp. GM60 PMI32_contig56.56, whole genome shotgun sequence.	AKJI01000049	NO	Lanthipeptide	EJM82825	response_regulator_containing_a_CheY-like	PMI32_02631	103139-103870	No		Gammaproteobacteria	Pseudomonadales	Pseudomonadaceae	Pseudomonas	Plant associated	
Pseudomonas sp. HPB0071 aczIi-supercont1.1.C12, whole genome shotgun sequence.	AQFP01000012	NO	AHL-Siderophore	ENA29726	hypothetical_protein	HMPREF1487_07980	284342-285055	Yes	ENA29724	Gammaproteobacteria	Pseudomonadales	Pseudomonadaceae	Pseudomonas	Human associated	
Pseudomonas sp. In5 P.sp_In5_contig_2, whole genome shotgun sequence.	LIRD01000002	NO	NRPS	KPN90385	hypothetical_protein	AL066_08565	893752-894354	Yes	KPN90383	Gammaproteobacteria	Pseudomonadales	Pseudomonadaceae	Pseudomonas	Environmental	Nunamycin
Pseudomonas sp. P482 P482.contig.1_19, whole genome shotgun sequence.	JHTS01000023	NO	Other	KDO00004	bacterial_regulatory_s,_luxR_family_protein	BV82_2016	14934-15695	No		Gammaproteobacteria	Pseudomonadales	Pseudomonadaceae	Pseudomonas	Plant associated	
Pseudomonas sp. Root562 contig_6, whole genome shotgun sequence.	LMGK01000023	NO	Lanthipeptide	KQZ81042	LuxR_family_transcriptional_regulator	ASD60_12055	113693-114424	No		Gammaproteobacteria	Pseudomonadales	Pseudomonadaceae	Pseudomonas	Plant associated	
Pseudomonas sp. VLB120, complete genome.	CP003961	YES	AHL-Thiopeptide	AGZ33361	LuxR_family_transcriptional_regulator	PVLB_02760	637195-637908	Yes	AGZ33362	Gammaproteobacteria	Pseudomonadales	Pseudomonadaceae	Pseudomonas	Environmental	
Pseudomonas syringae pv. apii strain ICMP2814 PapICMP2814_Contig_7, whole genome shotgun sequence.	LJPR01000034	NO	NRPS	KPW40335	Transcriptional_regulator,_LuxR_family,	ALO87_03678	85711-86475	No		Gammaproteobacteria	Pseudomonadales	Pseudomonadaceae	Pseudomonas	Plant associated	
Pseudomonas syringae pv. syringae strain ICMP3023 PsyICMP3023_Contig_27, whole genome shotgun sequence.	LJRK01000078	NO	AHL-PKS	KPY71543	Regulatory_protein_rhlR	ALO45_01493	26195-26908	Yes	KPY71497	Gammaproteobacteria	Pseudomonadales	Pseudomonadaceae	Pseudomonas	Plant associated	
Ralstonia sp. A12 Contig2, whole genome shotgun sequence.	JSZO01000002	NO	Other	KHK57885	LuxR_family_transcriptional_regulator	PI87_05485	243606-244361	No		Betaproteobacteria	Burkholderiales	Burkholderiaceae	Ralstonia	Environmental	
Rhizobium etli CFN 42 plasmid p42f, complete sequence.	CP000138	YES	AHL-NPRS	ABC94329	ranscriptional_regulator_(activator)_protei	RHE_PF00439	491883-492608	Yes	ABC94330	Alphaproteobacteria	Rhizobiales	Rhizobiaceae	Agrobacterium	Plant associated	
Rhizobium leguminosarum bv. trifolii WSM1689 plasmid, complete sequence.	CP007048	YES	AHL-bacteriocin	AHF88043	LuxR_family_transcriptional_regulator	RLEG3_02875	71235-71960	Yes	AHF88044	Alphaproteobacteria	Rhizobiales	Rhizobiaceae	Agrobacterium	Plant associated	
Rhizobium leguminosarum bv. trifolii WSM2012 genomic scaffold Rleg10scaffold_4, whole genome shotgun sequence.	JH719389	YES	AHL-NRPS	EJC75477	response_regulator_containing_a_CheY-like	Rleg10DRAFT_4093	586278-587000	Yes	EJC75476	Alphaproteobacteria	Rhizobiales	Rhizobiaceae	Agrobacterium	Plant associated	
Rhizobium sp. AAP43 AAP43_Contigs_45, whole genome shotgun sequence.	LJHS01000045	NO	AHL-Other	KPF41774	hypothetical_protein	IP76_19730	50935-51675	Yes	KPF41773	Alphaproteobacteria	Rhizobiales	Rhizobiaceae	Agrobacterium	Environmental	
Rhizobium sp. Root1212 contig_16, whole genome shotgun sequence.	LMDA01000008	NO	AHL-bacteriocin	KQV39924	hypothetical_protein	ASC86_22010	28649-29350	Yes	KQV39936	Alphaproteobacteria	Rhizobiales	Rhizobiaceae	Agrobacterium	Plant associated	
Rhizobium sp. Root149 contig_6, whole genome shotgun sequence.	LMGD01000023	NO	Thiopeptide	KQZ48532	DNA-binding_protein	ASD54_16845	117121-117852	No		Alphaproteobacteria	Rhizobiales	Rhizobiaceae	Agrobacterium	Plant associated	
Rhodopseudomonas palustris BisB18, complete genome.	CP000301	YES	AHL-NRPS	ABD87583	transcriptional_regulator,_LuxR_family	RPC_2028	2210961-2211689	Yes	ABD87584	Alphaproteobacteria	Rhizobiales	Bradyrhizobiaceae	Rhodopseudomonas	Environmental	
Roseivivax atlanticus strain 22II-s10s contig9, whole genome shotgun sequence.	AQQW01000009	NO	Ectoine	ETW11960	LuxR_family_transcriptional_regulator	ATO8_14877	147249-147992	No		Alphaproteobacteria	Rhodobacterales	Rhodobacteraceae	Roseivivax	Environmental	
Roseovarius tolerans strain EL-164 ROTO_contig000011, whole genome shotgun sequence.	LGVV01000011	NO	AHL-terpene	KNX42195	Transcriptional_activator_protein_LasR	ROTO_12350	38965-39666	Yes	KNX42196	Alphaproteobacteria	Rhodobacterales	Rhodobacteraceae	Roseovarius	Environmental	
erica subsp. enterica serovar Bovismorbificans genome assembly 4074_2#12, scaffold ERS008211SCcontig000002, whole genome sho	CTBG01000002	NO	Saccharide	CQB62135	DNA-binding_transcriptional_activator_SdiA	ERS008211_00535	173067-173789	No		Gammaproteobacteria	Enterobacterales	Enterobacteriaceae	Salmonella	Human assoicated	
Serratia marcescens SM39 DNA, complete genome.	AP013063	YES	AHL-NRPS	BAO36750	putative_quorum-sensing_LuxR-family	SM39_4837	5048939-5049655	Yes	BAO36751	Gammaproteobacteria	Enterobacterales	Yersiniaceae	Serratia	Human associated	
Serratia marcescens strain BWH57 aeviZ-supercont1.1.C4, whole genome shotgun sequence.	LETV01000004	NO	NRPS	KLX19829	hypothetical_protein	SK68_00902	204124-204843	No		Gammaproteobacteria	Enterobacterales	Yersiniaceae	Serratia	Human associated	
Serratia marcescens strain BWH57 aeviZ-supercont1.2.C14, whole genome shotgun sequence.	LETV01000014	NO	NRPS	KLX13910	LuxR_family_transcriptional_regulatory,	SK68_03785	965240-965968	No		Gammaproteobacteria	Enterobacterales	Yersiniaceae	Serratia	Human associated	
Serratia sp. ATCC 39006 ctg2, whole genome shotgun sequence.	AWXH01000002	NO	NRPS-PKS	ESN64571	hypothetical_protein	Ser39006_01979	1912449-1913201	No		Gammaproteobacteria	Enterobacterales	Yersiniaceae	Serratia	Environmental	
Serratia sp. ATCC 39006 ctg3, whole genome shotgun sequence.	AWXH01000003	NO	Betalactam	ESN62559	response_regulator_receiver_protein	Ser39006_03483	1029261-1029995	No		Gammaproteobacteria	Enterobacterales	Yersiniaceae	Serratia	Environmental	
Sinorhizobium americanum CCGM7 scaffold47, whole genome shotgun sequence.	JFGO01000047	NO	Thiopeptide	KEC77858	LuxR_family_transcriptional_regulator	SAMCCGM7_c4656	222156-222878	No		Alphaproteobacteria	Rhizobiales	Rhizobiaceae	Sinorhizobium	Plant associated	
Streptomyces canus telomycin biosynthetic gene cluster, complete sequence.	KP756960	YES	NRPS	AKQ13286	putative_LuxR_family_transcription_regulato	no_locus_tag	16169-16972	No		Actinobacteria	Streptomycetales	Streptomycetaceae	Streptomyces	Environmental	Telomycin
Thioclava sp. DT23-4 contig24, whole genome shotgun sequence.	AUNB01000023	NO	AHL-bacteriocin	KEO60115	hypothetical_protein	DT23_14585	42557-43165	Yes	KEO60116	Alphaproteobacteria	Rhodobacterales	Rhodobacteraceae	Thioclava	Environmental	
Tistrella mobilis KA081020-065 plasmid pTM3, complete sequence.	CP003239	YES	AHL-NRPS	AFK57307	LuxR_family_transcriptional_regulator	TMO_c0697	830445-831188	Yes	AFK57308	Alphaproteobacteria	Rhodospirillales	Rhodospirillaceae	Tistrella	Environmental	
Yersinia intermedia genome assembly 5139_2#5, scaffold ERS008476SCcontig000014, whole genome shotgun sequence.	CWJI01000014	NO	AHL-PKS	CRY56508	quorum-sensing_transcriptional_regulator	ERS008476_03552	49295-50029	Yes	CRY56509	Gammaproteobacteria	Enterobacterales	Yersiniaceae	Yersinia	Human associated	
Yersinia nurmii genome assembly 8016_2#91, scaffold ERS137967SCcontig000004, whole genome shotgun sequence.	CPYD01000004	NO	AHL-Siderophore	CNE38800	quorum-sensing_transcriptional_regulator	ERS137967_01435	172509-173252	Yes	CNE38846	Gammaproteobacteria	Enterobacterales	Yersiniaceae	Yersinia	Environmental	
Yersinia pestis KIM10+, complete genome.	AE009952	YES	AHL-Siderophore	AAM86925	putative_transcriptional_activator	y3375	3720096-3720839	Yes	AAM86924	Gammaproteobacteria	Enterobacterales	Yersiniaceae	Yersinia	Human associated	
